# Single-cell nanocapsules of gut microbiota facilitate fecal microbiota transplantation

**DOI:** 10.7150/thno.104852

**Published:** 2025-01-06

**Authors:** Weiliang Hou, Yuan Cao, Jifeng Wang, Fang Yin, Jiahui Wang, Ning Guo, Ziyi Wang, Xiaoqiong Lv, Chunlian Ma, Qiyi Chen, Rong Yang, Hong Wei, Juanjuan Li, Ruibing Wang, Huanlong Qin

**Affiliations:** 1 Research Institute of Intestinal Diseases, Shanghai Tenth People's Hospital Affiliated to Tongji University, 200072 Shanghai, China.; 2 Department of Gastroenterology, Shanghai Institute of Pancreatic Diseases, Changhai Hospital; National Key Laboratory of Immunity and Inflammation, Naval Medical University, 200433 Shanghai, China.; 3 Shanghai Cancer Institute, Renji Hospital School of Medicine, Shanghai Jiao Tong University, 200030 Shanghai, China.; 4 State Key Laboratory of Quality Research in Chinese Medicine, Institute of Chinese Medical Sciences, University of Macau, SAR 999078 Taipa Macau, China; 5 Institute of Clinical Science, Zhongshan Hospital, Fudan University, 200032 Shanghai, China; 6 Department of Pathology, Shanghai Tenth People's Hospital Affiliated to Tongji University, 200072 Shanghai, China.; 7 Intestinal Microenvironment Treatment Center, Shanghai Tenth People's Hospital Affiliated to Tongji University, 200072 Shanghai, China.; 8 Department of Pediatrics, Shanghai Tenth People's Hospital Affiliated to Tongji University, 200072 Shanghai, China.; 9 Central Laboratory, Clinical Medicine Scientific and Technical Innovation Park, Shanghai Tenth People's Hospital Affiliated to Tongji University, 200435 Shanghai, China.; 10 School of Life Sciences, Hainan University, 570228 Haikou, China.

**Keywords:** Single-cell nanocapsules, Microbial therapy, NanoFMT, Gut microbiota, Colitis

## Abstract

**Rationale:** Fecal microbiota transplantation (FMT) is advantageous for treating intractable diseases via the microbiota-gut-organ axis. However, invasive administration of gut microbiota via nasal feeding tubes limits the widespread application of FMT. Here, we attempted to develop a novel strategy to deliver gut microbiota using nanocapsules.

**Methods:** Single-cell nanocapsules were fabricated within 1 h by layer-by-layer assembly of silk fibroin and phosphatidylcholine to generate a protective nanoshell on the cell surface of complicated microbiota. The physical properties of the microbiota nanocapsules were analyzed. The protective effects of nanocapsules on the gastrointestinal tract were analyzed both *in vitro* and *in vivo*. The efficacy of FMT assisted by single-cell nanocapsules (NanoFMT) was evaluated using the inflammatory response, gut microbiota balance, and histopathological analysis in animal model.

**Results:** The nanocapsules achieved a good coating ratio for a single type of microbe and complex microbiota, resulting in a remarkable increase in the survival rate of microbes in the gastrointestinal tract. NanoFMT improved the diversity and abundance of the gut microbiota better than common FMT in germ-free mice. Moreover, NanoFMT alleviated intestinal inflammation and positively reversed the microbiota balance in a mouse model of colitis compared with common FMT, assisted by the inherent anti-inflammatory effects of silk fibroin and phosphatidylcholine.

**Conclusions:** Considering its rapid preparation, convenient delivery, and perfect therapeutic effect, we anticipate that NanoFMT may be a promising clinical candidate for next-generation FMT treatment.

## Introduction

Gut microbiota play a pivotal role in regulating human health in multiple aspects 1-3. Accordingly, fecal microbiota transplantation (FMT) has become a therapeutic strategy by transferring gut microbiota from a rigorously screened healthy donor to a diseased recipient for treating diseases such as diabetes mellitus, hypertension, inflammatory bowel disease (IBD), and obesity 4-7. Initially, microbiota slurries are prepared by homogenization and filtration of fecal material under sterile conditions, and then administered by invasive nasogastric feeding tubes or colonoscopy 8-10, which are not suitable for children and the elderly. Fecal microbiota capsules were hence developed by freeze-drying microbiota slurries for around 24 h at -80 ^°^C, which is suitable for oral administration 11-13. However, the complicated preparation procedure of microbiota capsules could decrease microbial activity and increase the manufacturing cost of microbiota products to a certain extent 14. Therefore, it is imperative to develop next-generation FMT technologies with concurrent oral convenience and high microbial activity.

Surface modifications have been used in microbial therapy to introduce exogenous functions 15-17. By coating the entire bacterial surface, nanocoated bacteria usually show improved bioavailability, a controlled release profile, and enhanced pharmaceutical effects compared with capsule packaging 18, 19. Recently, various coatings have been developed to increase bacterial survival and colonization *in vivo* 20-22. For instance, coating living bacteria with polydopamine nanoparticular immunosuppressants can enhance bacterial viability in the gastrointestinal tract, suppress hyperactive immune responses, and modulate the gut microbiome 23. Previous studies have mostly coated mono-microbes such as *Escherichia coli*, *Saccharomyces cerevisiae* (SC)*,* and* Listeria monocytogenes* 24-26. Coating thousands of types of microbiota with diverse surface structures is extremely challenging 27.

When used for FMT, rapid preparation, high activity, good protection, and low toxicity are required to coat the gut microbiota. However, only a few studies have met these criteria. Dopamine can polymerize on the surface of bacteria to achieve gastrointestinal protection but may decrease bacterial activity due to the high pH environment during polymerization 20. Silk fibroin can generate nanoshells by conformational transition from a random coil to a *β*-sheet under moderate conditions, while good protection requires up to four layers coating of silk fibroin 22. Here, we report a novel single-cell nanocapsule that coats the gut microbiota via a layer-by-layer self-assembly. Silk fibroin was self-assembled on the surface of the gut microbiota to generate nanoshells. Subsequently, phosphatidylcholine was used to reinforce the nanoshells by electrostatic adsorption. The nanocoating was completed within 1 h and achieved a high coating ratio of the gut microbiota, yet had no impact on the activity of the microbiota. Remarkable protection of nanocapsules was demonstrated *in vitro* and *in vivo*, resulting in improved abundance and diversity of the gut microbiota in germ-free (GF) mice after FMT. Excellent therapeutic efficacy of FMT assisted by single-cell nanocapsules (NanoFMT) was observed in a mouse model of colitis compared with that of regular FMT. Silk fibroin and phosphatidylcholine are approved by the Food and Drug Administration (FDA). Considering its clinical safety, convenient preparation, and perfect therapeutic effects, we anticipate that NanoFMT could innovate the current avenue of FMT to achieve more convenient and widespread clinical applications.

## Materials and Methods

### Materials and strains

*Escherichia coli* Nissle 1917 (EcN) was purchased from the China General Microbiological Culture Collection Center (CGMCC) and grown in Luria-Bertani (LB) medium at 37 °C with suitable antibiotics. *Pediococcus acidilactici* DQ2 (PA) was stored at the CGMCC (registration number: 7471) and grown in Man-Rogosa-Sharp medium. Cholic acid (CA), pepsin, and phosphatidylcholine were purchased from Adamas (Osaka, Japan). Dextran sulfate sodium (DSS) (reagent grade; MW 36-50 kDa) was purchased from Sangon.

### Preparation of silk fibroin

Silk fibroin solution was prepared as previously described 28. Briefly, cocoons were boiled in 0.02 M sodium bicarbonate for 20 min and then rinsed thoroughly using nanopure water. The extracted silk fibroin was dissolved in 9.3 M LiBr solution at 60 °C for 4 h, followed by the dialyzing against nanopure water using a Slide-a-Lyzer dialysis cassette (molecular weight cutoff 3500 Da, Pierce) at room temperature. The dialysate was centrifuged three times, each for 20 min, followed by filtering using a 0.4 μm glass-fiber syringe filter. The silk fibroin concentration was diluted to 0.1% (w/v) using nanopure water.

### Preparation of single-cell nanocoated microbes

Cultured EcN was washed three times using 0.01 M sodium phosphate buffer (pH 6.0) and centrifuged at 10000 rpm, followed by adding 0.1% (w/v) silk fibroin under shaking at 35 rpm for 10 min. The cells were then centrifuged, washed with nanopure water, resuspended in 0.1 M potassium phosphate buffer (pH 5.5), and incubated by vigorous shaking at 1000 rpm for 10 min. Silk fibroin-coated EcN was centrifuged, washed, resuspended in 5 mg/mL phosphatidylcholine, and incubated by vigorous shaking for 10 min. The coated cells were named as NanoEcN. PA and SC cells were coated using the aforementioned steps. The gut microbiota were obtained by collecting fresh mouse feces in sterile normal saline and filtering using a 70 μm pore nylon filter to remove large particulate and fibrous matter. The gut microbiota were successively coated with silk fibroin and phosphatidylcholine, and tentatively stored at 4 °C.

### Characterization of single-cell nanocoated microbes

Silk fibroin labeled with fluorescein isothiocyanate (FITC) and phosphatidylcholine labeled with rhodamine B on the surface of nanocoated microbial cells were observed using laser scanning confocal microscopy (Leica TCS SP8, Germany). The percentage of nanocoated microbial cells was examined using flow cytometry (Beckman CytoFlex). The morphology of the microbes was visualized using transmission electron microscopy (TEM) (HITACH, Japan) and atomic force microscopy (AFM) (Dimension FastScan Bio, Bruker).

### Resistance assessment *in vitro*

An equal amount of nude microbes or nanocoated microbes was resuspended in 1 mL medium containing simulated gastric fluid (SGF) with 10 g/L pepsin in 0.85% NaCl solution (HCl) for 2 h at 37 ℃, then diluted and spread onto an agar plate. Microbes were inoculated in simulated intestinal fluid (SIF) with trypsin in KH_2_PO_4_ solution, CA solution, or the corresponding culture media at 37 °C for the predetermined time points. The nude microbes and nanocoated microbes were stored at 4 ℃ for 7 days to evaluate the stability of nanocapsules using TEM images.

### Adhesion observation *in vitro*

The mouse colon was cut into small sections and incubated in 3% DSS for 48 h at 37 °C to simulate mucus injury. After 4′,6-diamidino-2-phenylindole dihydrochloride (DAPI) staining, EcN or NanoEcN (10^7^ colony forming units [CFU]/mL) was added under mild shaking for 20 min, followed by washing with PBS for imaging or colony counting.

### *In vitro* assessment of anti-inflammation properties

RAW 264.7 macrophage cells were cultured in 24-well plates (Dulbecco's modified eagle medium, DMEM) at 37 °C for 24 h and washed with PBS. Subsequently, silk fibroin or phosphatidylcholine was cultured with RAW 264.7 cells in the absence or presence of lipopolysaccharide (LPS) (1 μg/mL) for 24 h. The supernatant liquid was used for measuring NO content and cytokine levels, while cultured RAW 264.7 cells were labeled with an ROS probe (10 μM DCFH-DA) for flow analysis or confocal imaging, or stained with FITC-conjugated anti-CD86 antibody to analyzes M1 macrophage polarization by flow analysis.

### Cell safety evaluation

Caco-2 cells were seeded in 96-well plates (DMEM) at 37 °C for 24 h, washed with PBS, and incubated in a new DMEM containing phosphatidylcholine or silk fibroin solution for another 24 h. Thiazolyl blue (MTT) (0.5 mg/mL) was added to the medium under dark conditions for 1 h. The medium was then removed, and the wells were filled with dimethyl sulfoxide for absorbance measurement at 570 nm. Cell Counting Kit-8 (CCK8) was added to the medium under dark conditions for 1 h to measure the absorbance at 450 nm.

### Animals

SPF Balb/c mice (male, 6 weeks old) were purchased from Jiesijie Laboratory Animal Technology. GF KM mice (male, 6 weeks old) were obtained from the Department of Laboratory Animal Science of the Tenth People Hospital of Tongji University and bred in a gnotobiotic environment. All animal procedures complied with the Shanghai Medical Experimental Animal Care guidelines. Animal protocols were approved by the Institutional Animal Care and Use Committee of Shanghai Tenth People Hospital (SHDSYY-2021-6483).

### Oral bioavailability in SPF mice

Six-week-old male Balb/c mice were randomly divided into two groups (n = 6) and administered 2 × 10^7^ CFU/mouse EcN or NanoEcN via oral gavage. At 4 or 120 h post-administration, mice were sacrificed. The intestinal tracts were harvested and imaged using an *in vivo* imaging system (IVIS Lumina II, Caliper), and colonies were counted in plates with suitable antibiotics.

### Gut microbiota distribution in GF mice

Six-week-old male GF KM mice were randomly divided into two groups (n = 4) and treated with FMT (2 × 10^6^ CFU/mouse) or NanoFMT (2 × 10^6^ CFU/mouse). At 24 h post-administration, mice were sacrificed and fecal samples were collected for 16S rRNA gene sequencing. Total DNA was extracted from colonic contents and sequenced by building a sequencing library on an Illumina HiSeq 2500. The original image data files obtained using high-throughput sequencing were converted into sequenced reads using base-calling analysis. The results were stored in a FASTQ file. Data analysis was performed using BMKCloud to analyze species diversity, abundance, and community structure.

### *Salmonella typhimurium* (STm)-induced colitis model

A STm-induced colitis model was established as described previously 29. Balb/c mice (male, 6-8 weeks) were treated with 100 μl streptomycin solution (200 mg/mL) prior to infection by oral inoculation with 1 × 10^9^ CFU of *Salmonella*. Mice were randomly divided into three groups (n = 5) and treated with PBS, FMT (2 × 10^7^ CFU/mouse/day), or NanoFMT (2 × 10^7^ CFU/mouse/day) on days 2 and 4 post-infection. Mice were weighed daily and sacrificed on day 6 post-infection. The inflamed colons of mice were sampled for blinded histopathological analysis, and mouse blood was collected to prepare serum samples by centrifugation for cytokine level detection using ELISA kits.

### DSS-induced colitis model

Balb/c mice (male, 6-8 weeks) were fed 3% DSS salt in sterile drinking water for 7 days. Mice were randomly divided into three groups (n = 5) and administered PBS, FMT (2 × 10^7^ CFU/mouse/day), or NanoFMT (2 × 10^7^ CFU/mouse/day) via oral gavage for 5 days. On day 6, mice were orally administered 600 mg/kg mouse FITC-dextran. After 4 h, mice were sacrificed. Serum samples were used to measure cytokine levels using commercially available ELISA kits (MultiSciences Biotech, China), and intestinal permeability was measured by photometric analysis at an excitation wavelength of 4855 nm and an emission wavelength of 528 nm. The colons of mice were sampled for blinded histopathological analysis.

### *In vivo* biosafety assessment

Balb/c mice (male, 6-8 weeks) were randomly divided into two groups (n = 6) and orally administered PBS or NanoFMT (2 × 10^7^ CFU/mouse/day) daily. After 14 days, mice were sacrificed, and blood and organs were used for hematological and histopathological analyses, respectively.

### Histopathology analysis

Tissue samples were fixed in 4% formalin, embedded in paraffin according to standard procedures, and then sectioned at 4 μm for hematoxylin and eosin (H&E) and myeloperoxidase (MPO) staining. All tissue images were captured using a 3D HISTECH Pannoramic 250 (3DHISTECH, Hungary).

### Data analysis

Three independent samples were used for *in vitro* experiments, and at least four independent samples were used for animal experiments. We analyzed data using the IBM SPSS software, compared samples between groups using ANOVA, and made multiple comparisons using the least-significant difference method. Statistical significance was set at p < 0.05.

## Results

### Fabrication of single-cell nanocapsules

**Figure 1:**
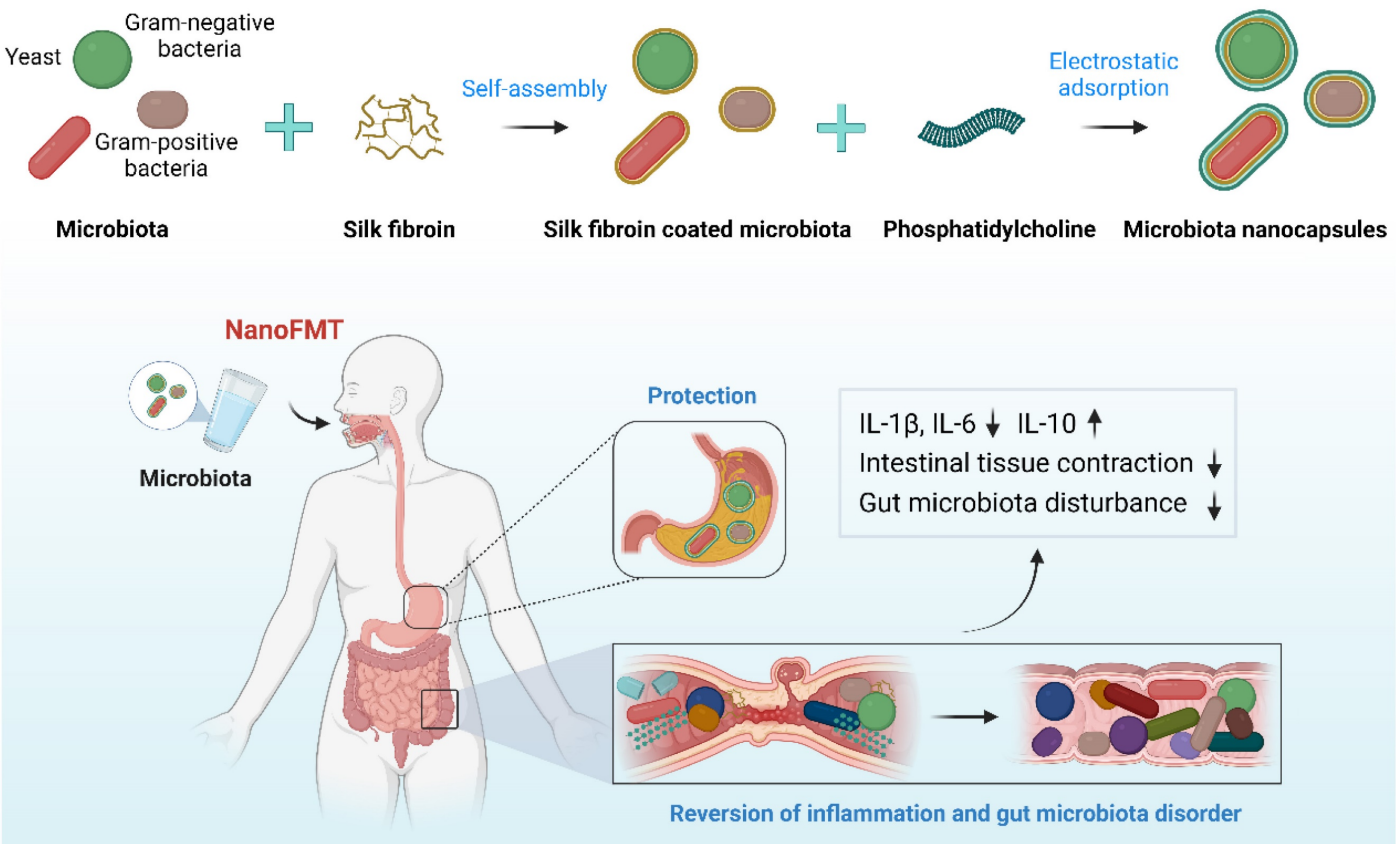
Schematic illustration of NanoFMT.

Silk fibroin, a natural polymer, has been approved by the FDA as a biomaterial owing to its biocompatibility, biodegradability, non-immunogenicity, and innate anti-inflammatory effects. Interestingly, silk fibroin can self-assemble into protective nanoshells on the surface of different nanoparticles transitioning from a random coil to a *β*-sheet conformation, which has been studied for potential drug delivery 30. We found that four layers of silk fibroin could achieve a good coating of microbes 22, which was too tedious for coating complex microbiota, perhaps causing a substantial loss of microbial activity. Phosphatidylcholine is largely responsible for establishing a protective hydrophobic surface and therefore plays a key role in mucosal defense 31. Owing to its zwitterionic nature, phosphatidylcholine may form nanoshells via the electrostatic adsorption of silk fibroin. Accordingly, we designed a layer-by-layer strategy to coat the gut microbiota with silk fibroin and phosphatidylcholine to prepare single-cell nanocapsules to improve the gastrointestinal survival of fecal microbiota (Figure 1).

**Figure 2:**
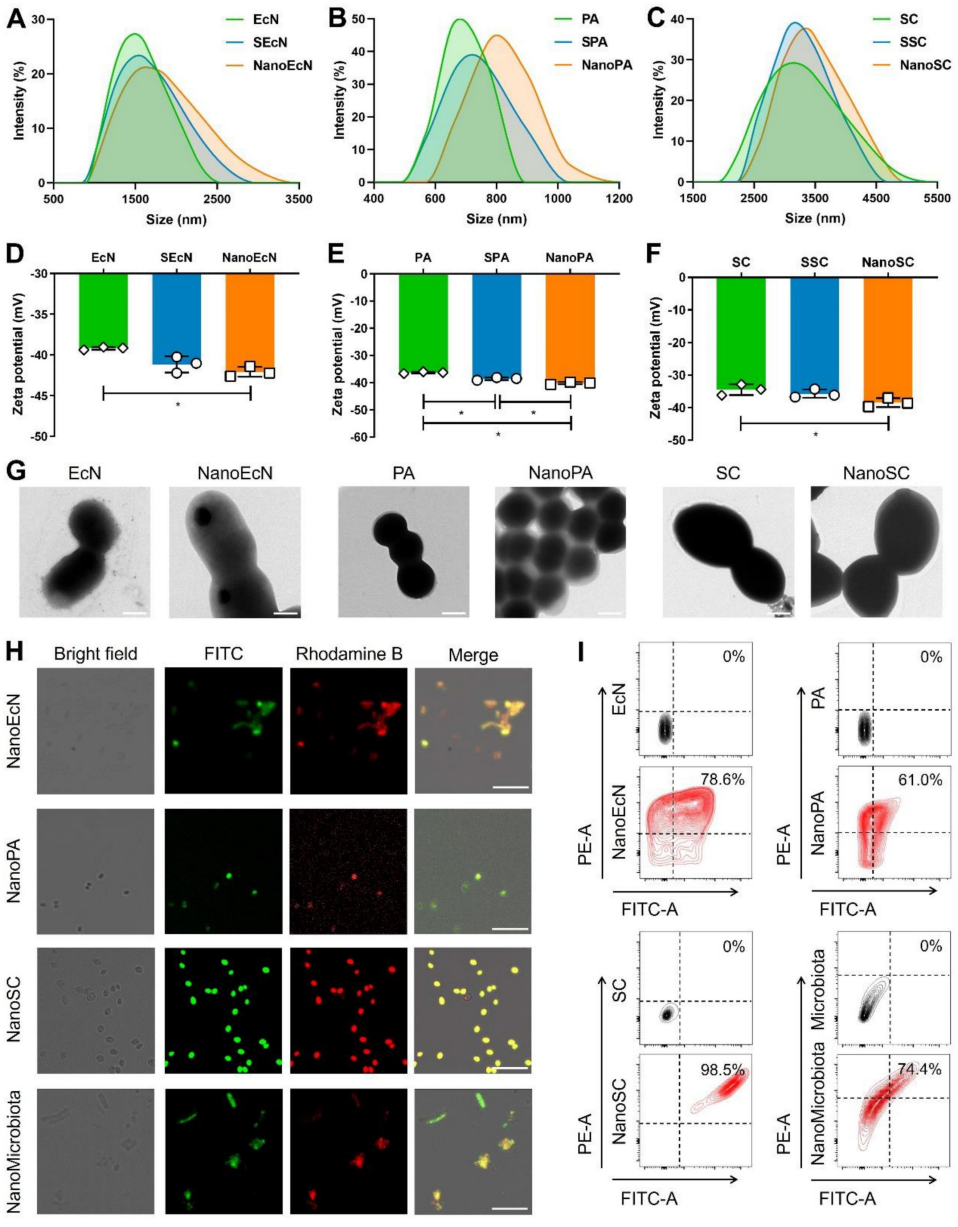
Preparation of single-cell nanocapsules of the gut microbiota. EcN, PA, SC, and microbiota were coated by co-incubation with 0.1% (w/v) silk fibroin while shaking at 35 rpm for 10 min before being resuspended in 0.1 M K^+^ phosphate buffer with vigorous vortexing for 10 min. Silk fibroin coated cells were centrifuged, washed, resuspended in 5 mg/mL phosphatidylcholine, and incubated with vigorous shaking for 10 min. The final coated cells were named NanoEcN, NanoPA, NanoSC, and NanoMicrobiota. (A-C) Size distribution of microbes and their nanoderivatives. (D-E) Zeta potentials of microbes and their nanoderivatives. (G) Representative TEM images of microbes and their nanoderivatives. Scale bar: 500 nm (EcN, NanoEcN, PA, and NanoPA) or 2 μm (SC and NanoSC). (H) Typical confocal images of microbes and their nanoderivatives. The green and red channels indicate silk fibroin labeled with FITC and phosphatidylcholine labeled with rhodamine B, respectively. Scale bar: 10 μm or 25 μm (NanoSC). (I) Flow cytometric analysis of microbes and their nanoderivatives.

The gut microbiota mainly include gram-negative bacteria, gram-positive bacteria, and fungi 32, whose cell walls present different structural features that may affect the nanocoating ratio of silk fibroin and phosphatidylcholine. Therefore, we first chose three representative probiotics to evaluate the feasibility of single-cell nanocapsules for coating microbiota, including the gram-negative bacteria EcN, gram-positive bacteria PA, and the fungus SC. Silk fibroin was prepared by extraction from cocoons in a boiling solution of 0.02 M sodium bicarbonate. The resulting silk fibroin was purified by stirring in 9.3 M LiBr at 60 °C for 4 h and subsequently dialyzed against nanopure water at room temperature. Microbes were simply shaken with 0.1% silk fibroin solution for 10 min to generate β-sheet conformation from a random coil and subsequently vigorously shaken in 0.1 M K^+^ phosphate buffer (pH 5.5) for an additional 10 min to stabilize the formed coating by a salting-out process 33. Silk fibroin-coated microbes were vigorously shaken in 5 mg/mL phosphatidylcholine for 10 min to form complete nanocoatings via electrostatic adsorption. Silk fibroin-coated microbes and phosphatidylcholine-coated microbes were renamed by adding the prefixes S and Nano, respectively (Figure S1). Dynamic light scattering showed that the particle size of microbes increased during the coating process (Figures 2A-C), whereas the zeta potential of microbes only slightly decreased because of the electronegative properties of silk fibroin and the zwitterionic nature of phosphatidylcholine (Figures 2D-F). The results indicate that the desired nanocoating could be obtained sequentially using the layer-by-layer strategy of silk fibroin and phosphatidylcholine. Subsequently, we observed morphological differences between the microbes and their nanoderivatives using TEM and AFM. A remarkable increase in the surface thickness and roughness of EcN after coating with silk fibroin and phosphatidylcholine was observed compared with that in nude EcN (Figure 2G and Figure S2). Similar morphological changes were also observed in PA, SC, and their nanoderivatives, demonstrating that single-cell nanocapsules can coat different types of microbes.

After successfully coating gram-negative bacteria, gram-positive bacteria, and fungi, we attempted to fabricate single-cell nanocapsules of complex gut microbiota using a layer-by-layer strategy. The gut microbiota were prepared by homogenization and filtration of mouse feces using a 70 μm pore nylon filter to remove large impurities. Fecal filtrates containing various microbiota were sequentially coated with silk fibroin and phosphatidylcholine. As the impurity interferences from the fecal filtrate (such as neutral fiber and negatively charged proteins) could impact charge measurement and make identification of the microscopic images from the nanocoated and nude microbiota difficult, we attempted to assess the coating efficacy of the gut microbiota by fluorescently labeling the nanoshells. Silk fibroin and phosphatidylcholine were labeled with FITC and rhodamine B, respectively. Confocal images showed that the fluorescence signals of FITC and rhodamine B sequentially merged during the coating process of a single type of microbe (Figure 2H and Figures S3A-C). Similar fluorescence images were obtained during the fabrication process of single-cell nanocapsules of gut microbiota (Figure 2H and Figure S3D), indicating that the layer-by-layer strategy could coat complex gut microbiota. Quantitative analysis by flow cytometry suggested that the coating ratio of microbiota could reach up to 74.4% using the layer-by-layer strategy, which was consistent with that of a single type of microbe (Figure 2I). Importantly, a high retrieval rate of microbes remained after decorations (Figure S4). Collectively, these results demonstrate that the layer-by-layer strategy using silk fibroin and phosphatidylcholine can achieve excellent coating to prepare single-cell nanocapsules of complex gut microbiota.

### Impact of nanocapsules on gastrointestinal tolerance, intestinal adhesion, and anti-inflammation *in vitro*

**Figure 3:**
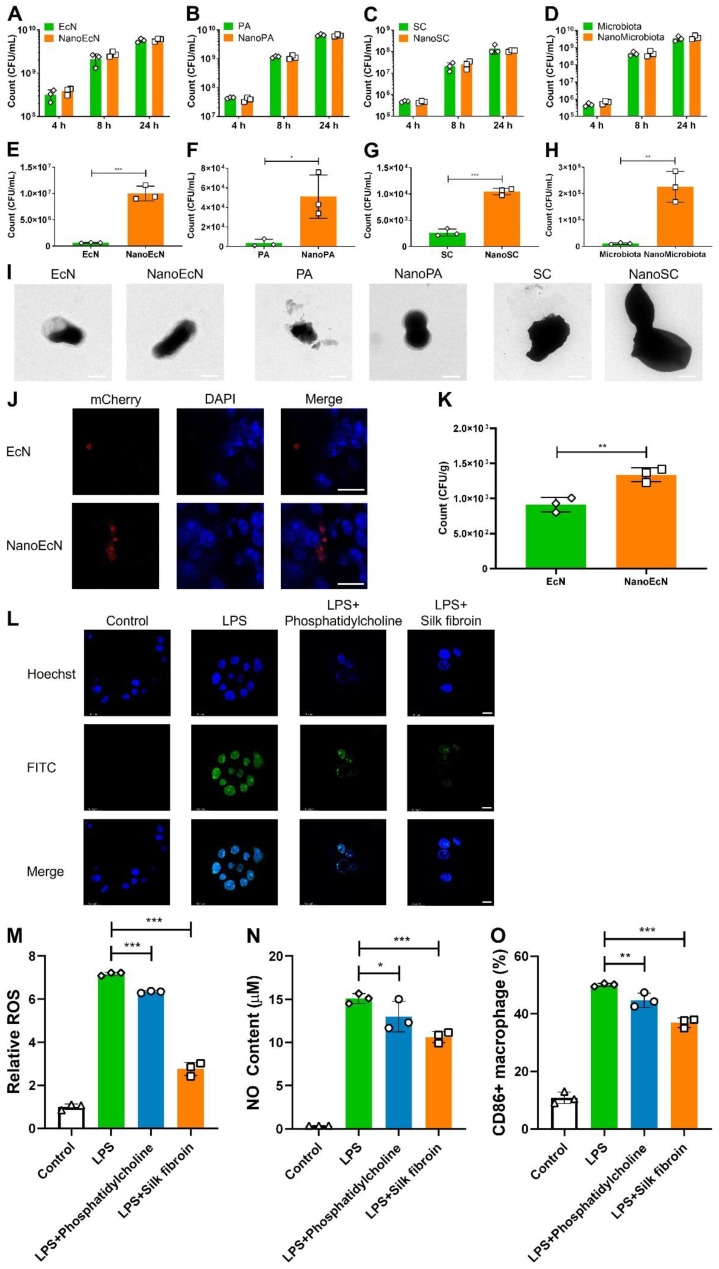
Impact of nanocapsules on resistance to environmental assaults, intestinal adhesion, and anti-inflammation. (A-D) Growth curves of nanocapsule-coated EcN, PA, SC, and microbiota at 37 ℃ and 200 rpm. (E-H) The survived number of nanocapsule-coated EcN, PA, SC, and microbiota after exposure to SGF (pH 2.5) supplemented with pepsin for 2 h at 37 ℃ and 200 rpm. (I) TEM images of nanocapsule-coated EcN, PA, SC, and microbiota after exposure to SGF for 2 h. (J, K) Typical confocal images (J) and bacterial count (K) of nanocapsule-coated EcN after adhesion to DSS-damaged intestines for 30 min. The blue and red channels indicate intestinal epithelial cells labeled with DAPI and EcN with mCherry fluorescence, respectively. Scale bar: 10 μm. (L-O) Anti-inflammatory properties of the nanocapsule constituents (silk fibroin and phosphatidylcholine) determined by analyzing ROS level (L, M), NO content (N), and percentage of CD86+ macrophages (O) in RAW 264.7 cells. The blue and green channels indicate RAW 264.7 cells labeled with DAPI and ROS labeled with a DCFH-DA probe, respectively. Scale bar: 10 μm. Error bars represent standard error of mean (n = 3). p< 0.05, *, p < 0.01, **, p < 0.001, ***.

One may speculate that nanocoating could potentially impact microbial bioactivity. We first measured the strain growth after the layer-by-layer coating. Interestingly, the growth of NanoEcN, NanoPA, NanoSC, and NanoMicrobiota was similar to that of nude ones, indicating that the layer-by-layer coating by silk fibroin and phosphatidylcholine did not disturb microbial growth, and the strains could proliferate by breaking the nanoshell (Figures 3A-D and Figure S5).

Most microbes die under acid attack and pepsin enzymolysis in the stomach after oral administration without nanoencapsulation. To investigate the protective effects of the nanocapsules on microbiota, we first examined the tolerance of nanocoated microbes in SGF with 10 g/L pepsin in 0.85% NaCl solution (HCl, pH 2.5). Equal quantities of nanocoated and nude microbes were incubated in SGF at pH 2.5 for 2 h. The survival of nanocoated microbes was significantly improved by an order of magnitude compared with that of the nude versions of EcN, PA, and SC, which was similar in SGF at different pHs (Figures 3E-G and Figures S6-7). In particular, the bacterial count of NanoMicrobiota increased 20.5-fold compared with that of the nude microbiota (Figure 3H). TEM images demonstrated that the nanocoated microbes maintained their morphological integrity, whereas the nude microbes were in a broken state (Figure 3I). Microbial growth was also evaluated in SIF. The nanocoated microbes presented a faster growth rate than the nude microbes in the early stage, which was consistent with the results in SIF with different trypsin concentrations (Figures S8-9). CA extracted from the intestinal tract can deactivate microbes. We evaluated the resistance of microbes to CA (0.3 mg/mL) and found that nanocapsules well-armed microbes with the highest activity up to 1.7 folds than the nude microbes (Figure S10). The advantage of nanocapsule-coated microbes was further demonstrated by an increase in CA concentration (Figure S11). Moreover, the nanocapsules exhibited good stability during long-term storage (Figure. S12). These results suggest that nanocapsules constructed using silk fibroin and phosphatidylcholine could potentially protect microbes from assault on the gastrointestinal tract.

Intestinal adhesion is a key factor that influences intestinal microbial colonization. We constructed a DSS-damaged colon to assess microbial adhesion to the inflamed intestine *in vitro*. Nanocapsules significantly improved intestinal adhesion of EcN, with a 1.5-fold increased count compared with the nude EcN after 30 min of co-incubation with the inflamed intestine (Figures 3J-K), perhaps due to the elevated particle size and surface area provided by the nanocoating.

Anti-inflammation is a critical pathway in the treatment of intestinal inflammation. We investigated the anti-inflammatory properties of the nanocapsule components, silk fibroin and phosphatidylcholine. RAW 264.7 macrophage cells were cultured in DMEM with 1 μg/mL LPS and silk fibroin or phosphatidylcholine for 24 h, then ROS level, NO content, and percentages of CD86+ macrophages were analyzed in RAW 264.7 cells. LPS induces the generation of ROS and NO, leading to macrophage polarization. We found that silk fibroin and phosphatidylcholine significantly weakened ROS generation, while silk fibroin achieved better ROS elimination with 2.6-fold reduction than LPS induction (Figures 3L-M and Figure S13A). Interestingly, silk fibroin and phosphatidylcholine also decreased NO content in RAW 264.7 cells after exposure to LPS (Figure 3N and Figure S13B). LPS induced M1 phenotype macrophage polarization in RAW 264.7 cells, which was inhibited by silk fibroin and phosphatidylcholine, as reflected by the decreased expression of CD86 (Figure 3O and Figure S13C) and the reversed cytokine levels (Figure S14). The cell toxicity of the nanocapsules was investigated in intestinal cells, and a significant decrease in cell viability was not found by the MTT and CCK8 methods in Caco-2 cells after exposure to silk fibroin and phosphatidylcholine (Figure S15). These results indicate that the nanocapsules provided additional anti-inflammatory effects and did not damage intestinal cells.

### Nanocapsules mediated the efficiency of intestinal colonization and FMT

**Figure 4:**
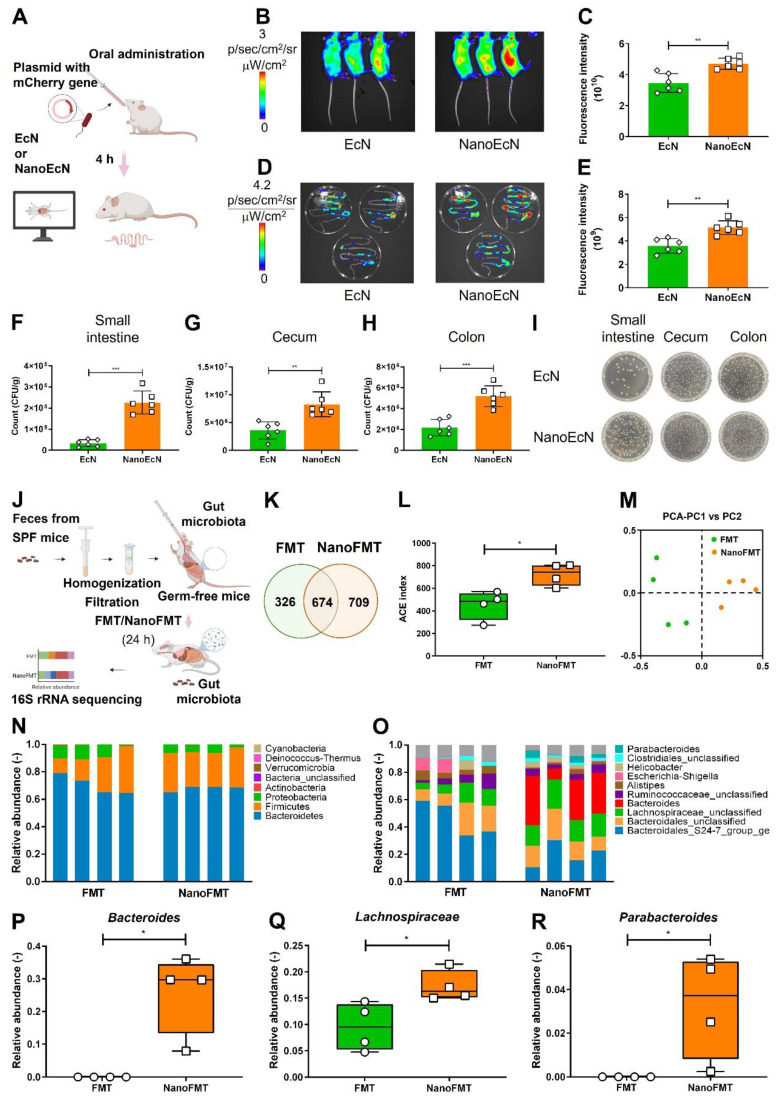
Nanocapsule-mediated intestinal colonization in SPF mice and gut microbiota distribution in germ-free mice. (A) Experimental design for evaluating *in vivo* resistance of NanoEcN against insults within the GI tract in SPF Balb/c mice. Mice were randomly divided into 2 groups (n = 6) and fed 2 × 10^7^ CFU/mouse EcN or NanoEcN via oral gavage. (B) Typical images captured using IVIS after 4 h post-administration. (C) Quantification of fluorescence intensities in mice. Error bars represent standard error of mean. (D) Representative fluorescent images of the GI tract. (E) Quantification of fluorescence intensities of the GI tract. (F-H) Bacterial count of EcN in small intestine (F), cecum (G) and colon (H). (I) Type images of EcN colony in the small intestine, cecum, and colon. (J) Experimental design of FMT to evaluate gut microbiota distribution in germ-free mice. Mice were randomly divided into 2 groups (n = 4) and treated with FMT (2 × 10^6^ CFU/mouse) or NanoFMT (2 × 10^6^ CFU/mouse). (K) Venn diagram of the gut microbiota after 24 h of NanoFMT treatment. (L) The ACE index represents alpha diversity analysis of the gut microbiota. Error bars represent standard error of mean. (M) The PCA index represents beta diversity analysis of the gut microbiota. (N, O) Abundance of phyla (N) and genera (O) in the gut microbiota. (P-R) Abundance of *Bacteroides* (P), *Lachnospiraceae* (Q), and *Parabacteroides* (R) in gut microbiota. p< 0.05, *, p < 0.01, **, p < 0.001, ***.

We further investigated the protection of microbes by nanocapsules in the gastrointestinal tract. Red fluorescent EcN was used as a model microbe to monitor bacterial intestinal engraftment (Figure 4A). Mice were orally administered EcN and sacrificed for imaging at 4 h post-administration. The fluorescence intensity of EcN was imaged using IVIS. Compared with nude EcN, nanocoated EcN accumulated more in the abdominal region (Figures 4B-C). Anatomic analysis showed that EcN was well distributed in the intestine and cecum of mice treated with the nanocoated EcN than that in nude EcN (Figures 4D-E). Interestingly, the increased counts of EcN by nanocapsules reached to 8.9 folds in the small intestine, 3.1 folds in the cecum and 2.4 folds in the colon compared with those of nude EcN (Figures 4F-I), indicating that nanocapsules significantly improved the intestinal engraftment of EcN. Furthermore, the colonization of nanocapsule-coated EcN presented a great advantage over that of EcN in the gastrointestinal tract after oral administration on day 5 (Figure S16).

To further investigate the impact of nanocoating on gut microbiota engraftment, we prepared NanoMicrobiota using microbiota isolated from healthy mice. Nude microbiota and NanoMicrobiota were orally administered into GF mice for FMT and NanoFMT, respectively. Mouse feces were collected on 24 h after gavage and analyzed using 16S ribosomal RNA gene sequencing (Figure 4J). A Venn diagram showed that 674 operational taxonomic units (OTUs) were shared by the NanoFMT and FMT groups, whereas the specific OTUs from the NanoFMT group increased by 383 compared with those from the FMT group, indicating that nanocoating significantly improved the survival rate of the gut microbiota in mice (Figure 4K). The NanoFMT group presented higher alpha diversity and significantly separated beta diversity than the FMT group (Figures 4L-M). The relative abundance of the gut microbiota was uniform at the phylum and genus levels between samples in the NanoFMT group, indicating better repeatability of FMT by the nanocapsules (Figures 4N-O and Figure S17). *Bacteroides*, a keystone species in the gut, was 14.5 thousand folds higher in the NanoFMT group than in the FMT group (Figure 4P). *Lactobacillus,* associated with recovery from IBD, showed a much higher relative abundance in the NanoFMT group 34, indicating that better inflammatory reversion could be achieved by NanoFMT (Figure 4Q). *Parabacteroides* have the physiological characteristics of carbohydrate metabolism and secrete short-chain fatty acids 35, which only survived in the NanoFMT group (Figure 4R). Collectively, these results indicate that nanocapsules can significantly ameliorate the resistance of microbes to the gastrointestinal tract microenvironment, thereby increasing the overall microbial survival rate and microbiota diversity.

### NanoFMT for regulating the gut microbiota and reversing intestinal inflammation

**Figure 5:**
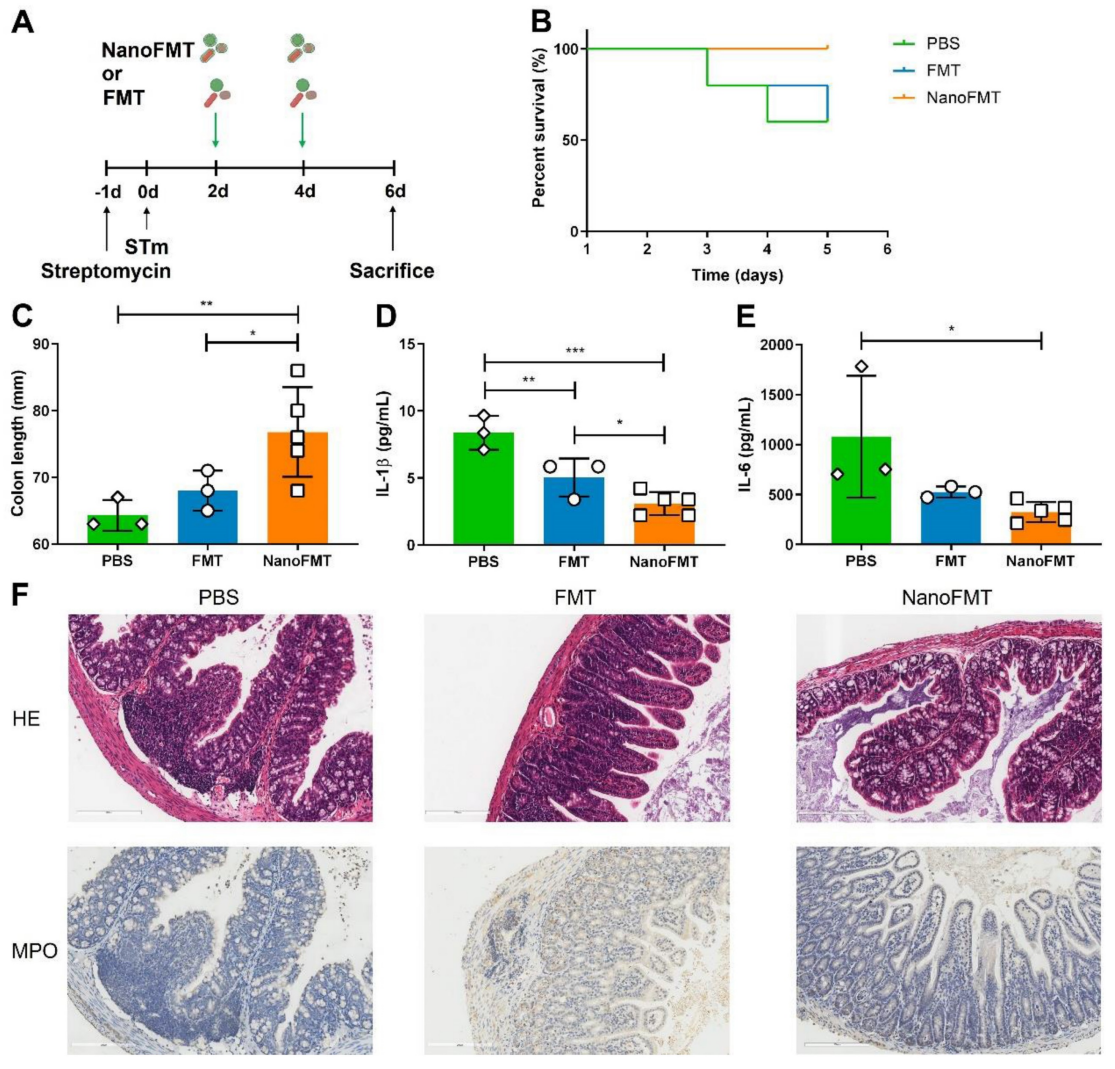
Therapeutic effect of NanoFMT in an STm-induced colitis mouse model. (A) Experimental design for the treatment of STm-induced colitis in mice. Mice were treated with 100 μl of streptomycin solution (200 mg/mL) prior to infection by oral inoculation with 1 × 10^9^ CFU of *Salmonella*. Mice were randomly divided into 3 groups (n = 5) and treated with PBS, FMT (2 × 10^7^ CFU/mouse/day), and NanoFMT (2 × 10^7^ CFU/mouse/day) on days 2 and 4 post-infection. All mice were sacrificed for sampling on day 6 post-infection. (B) Survival rate of mice during treatment. (C) Colon length after treatment. (D, E) Serum cytokine levels measured using commercially available ELISA kits including IL-1β (D) and IL-6 (E). (F) Histopathological images of H&E and MPO stained colon sections. Error bars represent standard error of mean (n = 5). p< 0.05, *, p < 0.01, **, p < 0.001, ***.

We subsequently examined the therapeutic effects of NanoFMT in an STm-induced colitis mouse model. Mice were administered streptomycin one day before STm infection and then orally administered gut microbiota nanocapsules for NanoFMT on days 2 and 4 post-infection. Common FMT and PBS treatments were used as the control and blank, respectively. Subsequently, mice were sacrificed on day 5 post-infection to analyze intestinal tissues and serum (Figure 5A). Some mice in the PBS group died on days 3 and 4 post-infection, while the death rate of the FMT group was relatively lower but still reached the same mortality rate as that of the PBS group on day 5 post-infection (Figure 5B). Conversely, no mice in the NanoFMT group died, indicating that NanoFMT had the best therapeutic effect. Therefore, we investigated the specific mechanism of NanoFMT in treating STm-induced colitis. STm infection can cause severe inflammation of the intestinal tract, thereby shortening the intestinal length 36. FMT alleviated inflammation and recovered the intestinal length to a certain extent, whereas the NanoFMT group exhibited the longest colon length (Figure 5C and Figure S18). Cytokine levels were determined using ELISA. Remarkably, NanoFMT reversed the cytokine levels of interleukin-1β (IL-1β) and IL-6 in the serum, in a more pronounced manner than FMT, indicating the superior improvement of systemic inflammation by NanoFMT (Figures 5D-E). Previous studies have demonstrated that silk fibroin and phosphatidylcholine exhibit inherent anti-inflammatory effects in animal models and clinical applications 37. Nanocapsules formed by silk fibroin and phosphatidylcholine can moderate intestinal inflammation, except providing gastrointestinal protection. Histopathological analysis of colon tissue was performed using H&E and MPO staining (Figure 5F). As expected, NanoFMT mitigated inflammation and hemorrhage in the colon and significantly fewer MPO-positive cells were observed in colon lesions in the NanoFMT group than in the FMT group.

**Figure 6:**
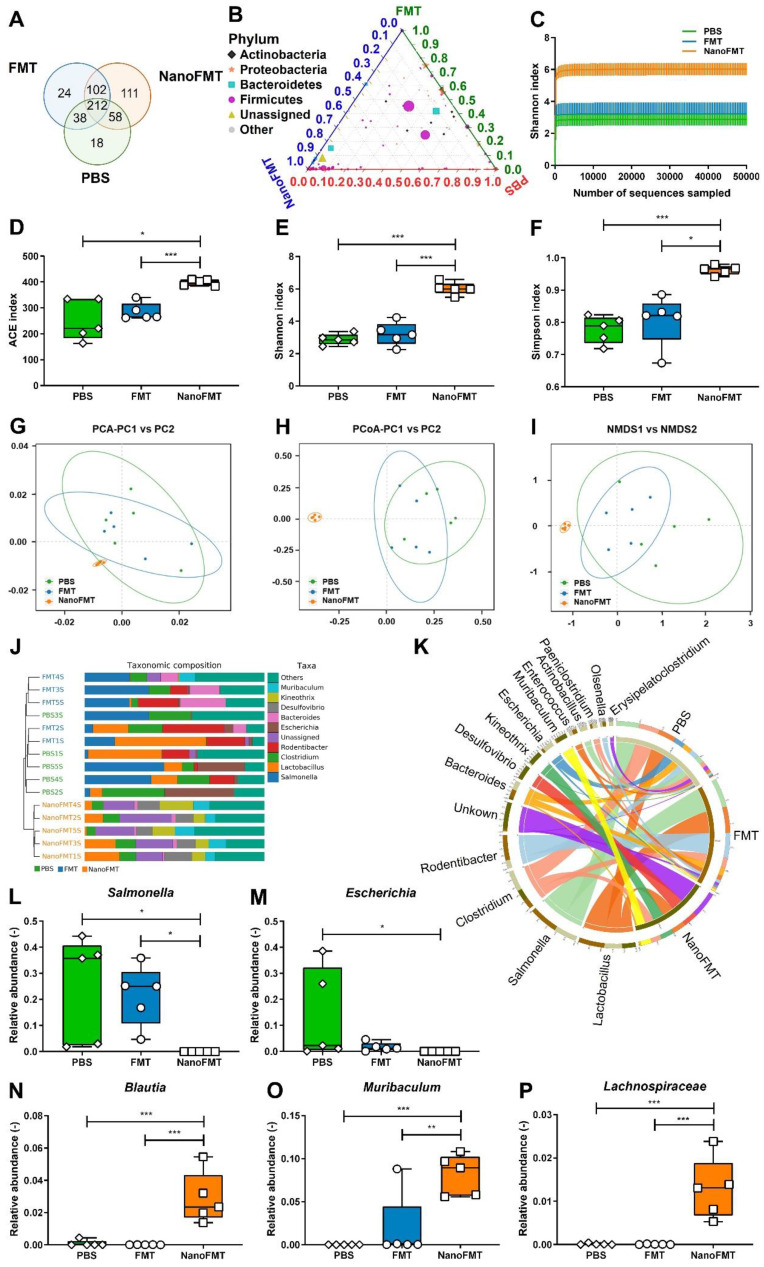
Gut microbiota analysis after the treatment of STm-induced mouse model of colitis using NanoFMT. (A) Venn diagram at the OTU level. (B) Ternary phase diagram at the genus level. (C) Shannon index curve. (D-F) Alpha diversity analysis of gut microbiota presented by the ACE (D), Shannon (E), and Simpson index (F). (G-I) Beta diversity analysis of gut microbiota presented by PCA (G), PCoA (H), and NMDS analysis (I). (J) UPGMA clustering tree with taxonomic composition. (K) Circos diagram of the microbial composition at the genus level. (L-P) Abundance of *Salmonella* (L), *Escherichia* (M), *Blautia* (N), *Muribaculum* (O), and *Ruminococcus* (P). Error bars represent standard error of mean (n = 5). p< 0.05, *, p < 0.01, **, p < 0.001, ***.

As NanoFMT markedly ameliorated the gut microbiota of GF mice (Figure 4), we further analyzed the effect of NanoFMT on the gut microbiota in STm-induced colitis. Venn diagrams showed 212 shared OTUs among the three groups (Figure 6A). There were 111 unique OTUs in the NanoFMT group, which was remarkably higher than 24 in the FMT group and 18 in the PBS group. The ternary phase diagram describes the ratio relationship of different attributes in the three groups, demonstrating a much higher relative abundance of the gut microbiota in the NanoFMT group than in the other two groups (Figure 6B). The Shannon index rarefaction curve shows the microbial diversity analyzed using different numbers of sequences. The highest Shannon index indicated the highest species diversity in the NanoFMT group (Figure 6C). No significant difference in alpha diversity was found between the PBS and FMT groups; however, greater alpha diversity was observed in the NanoFMT group (Figures 6D-F). Beta diversity analysis showed that the microbiota profiles of the NanoFMT group were distinctly different from those of the other groups (Figures 6G-I). The cluster tree showed similarities between the samples. Samples from the FMT and PBS groups presented some similarity, whereas both were evidently different from the samples from the NanoFMT group (Figure 6J). Significantly, *Salmonella* was present in each sample from the FMT and PBS groups, whereas it was nearly extinct in the NanoFMT group (Figures 6K-L and Figure S19). These results indicated that NanoFMT more thoroughly obliterated the infection source *Salmonella*. *Escherichia*, a highly virulent pathogen in *Proteobacteria* 38, was not found in the NanoFMT group but was heavily enriched in the PBS group (Figure 6M). In contrast, beneficial bacteria were enriched in the NanoFMT group. *Blautia* decreases inflammatory cytokine levels 39. *Mucispirillum* is responsible for repairing the intestinal mucosa by colonizing the colon 40. The relative abundance of these bacteria significantly increased in the NanoFMT group relative to that in the FMT and PBS groups (Figures 6N-O). Consistent with the GF mouse experiment, the relative abundance of *Lachnospiraceae* in the NanoFMT group was 205.0 and 118.2 folds higher than that in the PBS and FMT groups, respectively (Figure 6P). The BugBase phenotype prediction presented the relative abundance of aerobic, facultative anaerobic, anaerobic, biofilm-forming, and potentially pathogenic bacteria (Figure S20). Biofilm formation is an important characteristic of pathogenic bacteria 41. The highest relative abundance of potentially pathogenic and biofilm-forming bacteria was found in the PBS group, whereas the NanoFMT group presented the lowest relative abundance (Figure S21). These results demonstrate the excellent regulation of the gut microbiota by NanoFMT.

**Figure 7:**
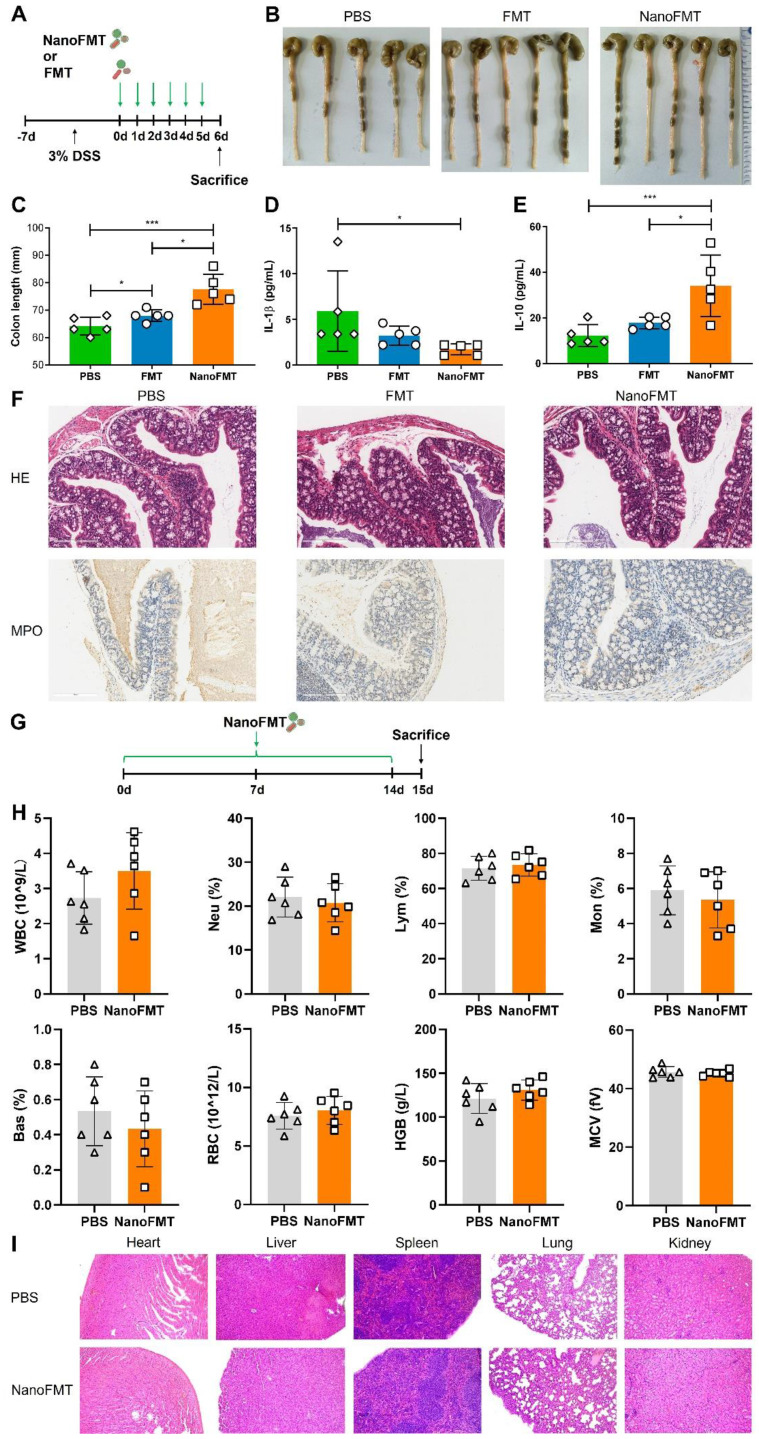
Therapeutic effect of NanoFMT in a DSS-induced mouse model of colitis and safety evaluation. (A) Experimental design for the treatment of DSS-induced colitis. Mice were fed 3% DSS salt in sterile drinking water for 7 days. Mice were randomly divided into 3 groups (n = 5) and then administered PBS, FMT (2 × 10^7^ CFU/mouse/day), or NanoFMT (2 × 10^7^ CFU/mouse/day) via gavage for 5 days. All mice were sacrificed for sampling on day 6 post-infection. (B) Photographs of colons sectioned from the treated mice. (C) Colon length after treatment. (D, E) Serum cytokine levels measured using commercially available ELISA kits including IL-1β (D) and IL-10 (E). (F) Histopathology images of H&E and MPO staining in the colon. Error bars represent standard error of mean (n = 5). (G) Experimental design for biosafety evaluation. Mice were randomly divided into 2 groups (n = 6), and orally administrated PBS or NanoFMT (2 × 10^7^ CFU/mouse/day) daily for 14 days, and then sacrificed for pathological analysis. (H) Hematological analysis of mice after biosafety evaluation. (I) Represented H&E images of the heart, liver, spleen, lungs, and kidney after biosafety evaluation. Error bars represent standard error of mean. p< 0.05, *, p < 0.01, **, p < 0.001, ***.

To verify the general applicability of NanoFMT in IBD, we evaluated its therapeutic effects in DSS-induced colitis. Mice were fed 3% DSS for 7 days to develop colitis, and then orally administered gut microbiota nanocapsules or nude gut microbiota daily for 5 days, and subsequently sacrificed to obtain intestinal tissues and serum (Figure 7A). Colon length was significantly prolonged by NanoFMT treatment, indicating that NanoFMT alleviated inflammation-induced tissue contraction in a more pronounced manner than FMT (Figures 7B-C). Furthermore, NanoFMT showed superior performance in reversing IL-1β and IL-10 levels (Figures 7D-E). Intestinal permeability was also ameliorated by NanoFMT (Figure S22), which could be a result of the combined effects of the modulated gut microbiota and reversed inflammation. H&E and MPO staining demonstrated that NanoFMT mitigated inflammatory responses and edema and reduced MPO-positive cells in the intestine, with the best effect among all the treatment groups (Figure 7F). Therefore, NanoFMT could be used as an all-purpose strategy to replace the current FMT for treating IBD.

Finally, the biosafety of NanoFMT was assessed (Figure 7G). Mice were orally administered nanocapsule-protected microbiota daily for 14 days. Subsequently, blood and organs were harvested for hematological and histopathological analyses (Figures 7H-I and Figure S23). Interestingly, no evident differences were found between the NanoFMT and control groups, indicating that the *in vivo* toxicity of NanoFMT could be neglected.

## Discussion

The reversal of gut microbiota dysbiosis and remission of intrinsic inflammation are critical for the treatment of IBD 42. Current therapeutics, including drugs and microbes, have an unmet need for effective treatment of IBD 43. In this study, we proposed a novel NanoFMT based on microbiota regulation and anti-inflammatory effects. In this system, nanocapsules were first constructed using silk fibroin and phosphatidylcholine and were well coated on the surface of single microbes, including gram-negative bacteria, gram-positive bacteria, and fungi (Figure 1). Interestingly, the gut microbiota nanocapsules were successfully established. Nanocapsules significantly ameliorated the gastrointestinal tolerance of the gut microbiota and intestinal adhesion and concurrently rendered extra anti-inflammatory effects, but did not affect the growth and proliferation of microbes (Figure 2). Compared with FMT, NanoFMT assisted by nanocapsules greatly improved the diversity and abundance of the gut microbiota in GF mice and eliminated pathogenic bacteria in a mouse model of colitis, which mainly benefited from the protection of nanocapsules (Figures 3 and 6). Interestingly, typical beneficial bacteria also increased in the NanoFMT group compared with those in the FMT group, perhaps contributing to the microbiota balance and inflammation reduction. The efficacy of NanoFMT was demonstrated in various models of colitis. Importantly, both silk fibroin and phosphatidylcholine are FDA-approved materials. Silk fibroin, a strong antioxidant, can eliminate ROS by own β-sheet structures and corresponding peptides, while phosphatidylcholine can inhibit inflammation by protecting the integrity and function of cell members 44, 45. Therefore, silk and phosphatidylcholine adjusted ROS and NO levels in macrophages (Figure 3) and mitigated intestinal inflammation in mice (Figures 5 and 7). In brief, NanoFMT, as an effective and safe therapy, presented good clinical prospects for IBD treatment, assisted by the inherent anti-inflammatory properties of nanocapsule components.

Recent studies have indicated that FMT has unique advantages in the treatment of refractory, recurrent, and chronic diseases, such as *Clostridium difficile* infection 46. In 2021, *Science* reported that PD-1 therapy responder-derived FMT, together with anti-PD-1, was used to treat patients with PD-1-refractory melanoma, presenting clinical benefits in 6 of 15 patients 7. In another clinical trial, FMT was used to decolonize multidrug-resistant organisms in renal transplant recipients. Participants treated with FMT had a longer time to recurrent infection than the controls who were not treated with FMT 47. These studies confirmed the promising application of FMT in the treatment of various diseases. Gastrointestinal tolerance and intestinal adhesion of NanoFMT was superior to common FMT, perhaps achieving better clinical outcomes. However, many challenges still need to be addressed before clinical application, such as the stability of the microbiota during long-term storage and establishment of a standardized production process.

## Conclusion

In summary, we report a new FMT technology based on nanomaterials in the form of NanoFMT to improve the delivery style of the current FMT for IBD. Silk fibroin and phosphatidylcholine rapidly formed nanoshells via a layer-by-layer approach on each cell of the gut microbiota to fabricate single-cell nanocapsules. Single-cell microbiota nanocapsules showed protective effects against the gastrointestinal environment during oral administration, achieving excellent delivery efficiency into the intestines without affecting microbiota proliferation.

NanoFMT presented better alpha and beta diversity of the microbiota structure in GF mice and superior anti-inflammatory activity in a mouse model of colitis than common FMT. Compared with the invasive administration of microbiota slurries and the cumbersome preparation of capsules in the current FMT, NanoFMT manifested oral convenience, ease of preparation, and excellent efficacy. Overall, this study provides a safe and novel strategy for improving current FMT treatment, portending great clinical application prospects.

## Supplementary Material

Supplementary figures.

## Abbreviations

AFM: Atom force microscopy
CA: Cholic acid
CCK8: Cell counting Kit-8
CFU: Colony forming units
CGMCC: China General Microbiological Culture Collection Center
DSS: Dextran sulphate sodium
DAPI: 4′,6-diamidino-2-phenylindole dihydrochloride
DMEM: Dulbecco's modified eagle medium
EcN: *Escherichia coli* Nissle 1917
FDA: Food and Drug Administration
FITC: Fluorescein isothiocyanate
FMT: Fecal microbiota transplantation
GF: Germ-free
H&E: Hematoxylin and eosin
IBD: Inflammatory bowel disease
IVIS: *In vivo* imaging system
LPS: Lipopolysaccharide
MPO: Myeloperoxidase
MTT: Thiazolyl blue
NanoEcN: Silk fibroin and phosphatidylcholine coated *Escherichia coli* Nissle 1917
NanoFMT: Fecal microbiota transplantation assisted by single-cell nanocapsules
OTUs: Operational taxonomic units
PA: *Pediococcus acidilactici*
SC: *Saccharomyces cerevisiae*
SGF: Simulated gastric fluid
SIF: Simulated intestinal fluid
STm: *Salmonella typhimurium*
TEM: Transmission electron microscopy
