# Supplementary material for: Single-cell nanocapsules of gut microbiota facilitate fecal microbiota transplantation
Source: Theranostics. 2025 Jan 6;15(5):2069–84. doi: 10.7150/thno.104852 (PMC11780513; doi:10.7150/thno.104852)
Supplement: Supplementary file 1 — Supplementary figures. [file thnov15p2069s1.pdf]

Single-cell nanocapsules of gut microbiota facilitate fecal microbiota transplantation

Weiliang Hou[1,2,3,4✉], Yuan Cao[5,6], Jifeng Wang[6], Fang Yin[1], Jiahui Wang[1], Ning Guo[1], Ziyi Wang[4], Xiaoqiong Lv[7], Chunlian Ma[7], Qiyi Chen[7], Rong Yang[8], Hong Wei[9], Juanjuan Li[10✉], Ruibing Wang[4✉], Huanlong Qin[1✉]

[1]Research Institute of Intestinal Diseases, Shanghai Tenth People's Hospital Affiliated to Tongji University, 200072 Shanghai, China.

[2]Department of Gastroenterology, Shanghai Institute of Pancreatic Diseases, Changhai Hospital; National Key Laboratory of Immunity and Inflammation, Naval Medical University, 200433 Shanghai, China.

[3]Shanghai Cancer Institute, Renji Hospital School of Medicine, Shanghai Jiao Tong University, 200030 Shanghai, China.

[4]State Key Laboratory of Quality Research in Chinese Medicine, Institute of Chinese Medical Sciences, University of Macau, SAR 999078 Taipa Macau, China

[5]Institute of Clinical Science, Zhongshan Hospital, Fudan University, 200032 Shanghai, China

[6]Department of Pathology, Shanghai Tenth People's Hospital Affiliated to Tongji University, 200072 Shanghai, China.

[7]Intestinal Microenvironment Treatment Center, Shanghai Tenth People's Hospital Affiliated to Tongji University, 200072 Shanghai, China.

[8]Department of Pediatrics, Shanghai Tenth People's Hospital Affiliated to Tongji University, 200072 Shanghai, China.

[9]Central Laboratory, Clinical Medicine Scientific and Technical Innovation Park, Shanghai Tenth People's Hospital Affiliated to Tongji University, 200435 Shanghai, China.

[10]School of Life Sciences, Hainan University, 570228 Haikou, China.

First Authors: Weiliang Hou, Yuan Cao, Jifeng Wang and Fang Yin contributed equally

to this work.

✉ Corresponding authors: Weiliang Hou (houweiliang@tongji.edu.cn) or Juanjuan Li (lijuanjuan@hainanu.edu.cn) or Ruibing Wang (rwang@um.edu.mo), or Huanlong Qin (huanlong_qin@live.cn).

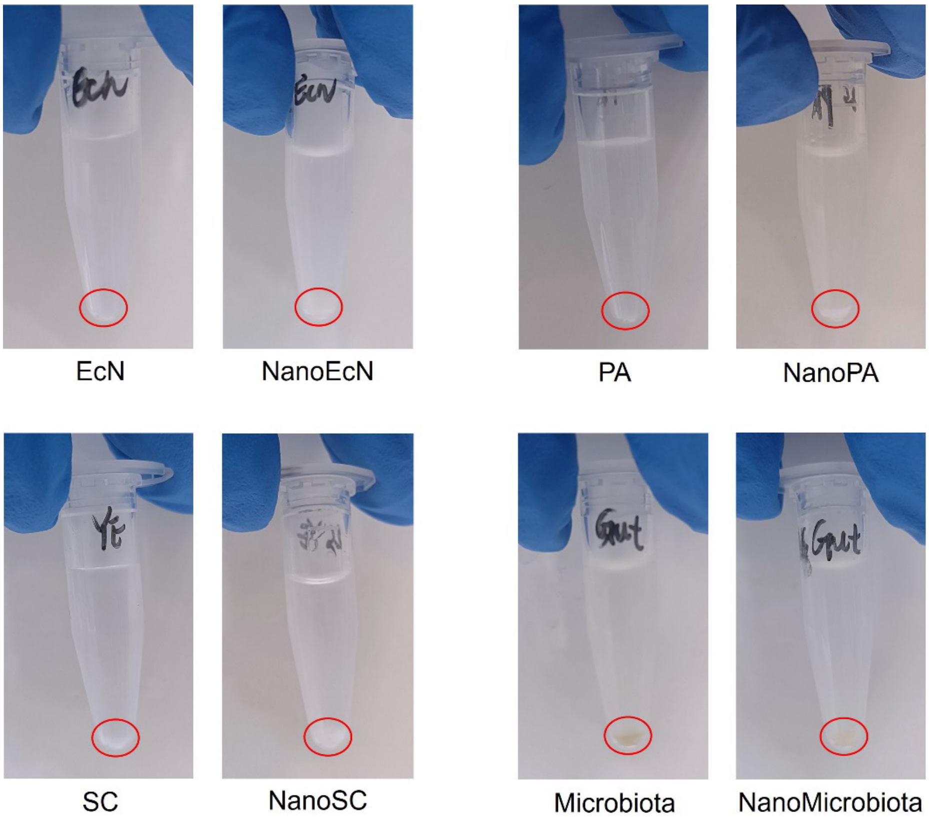

Figure S1 Morphologic change of microbes after nanocapsule-coating.

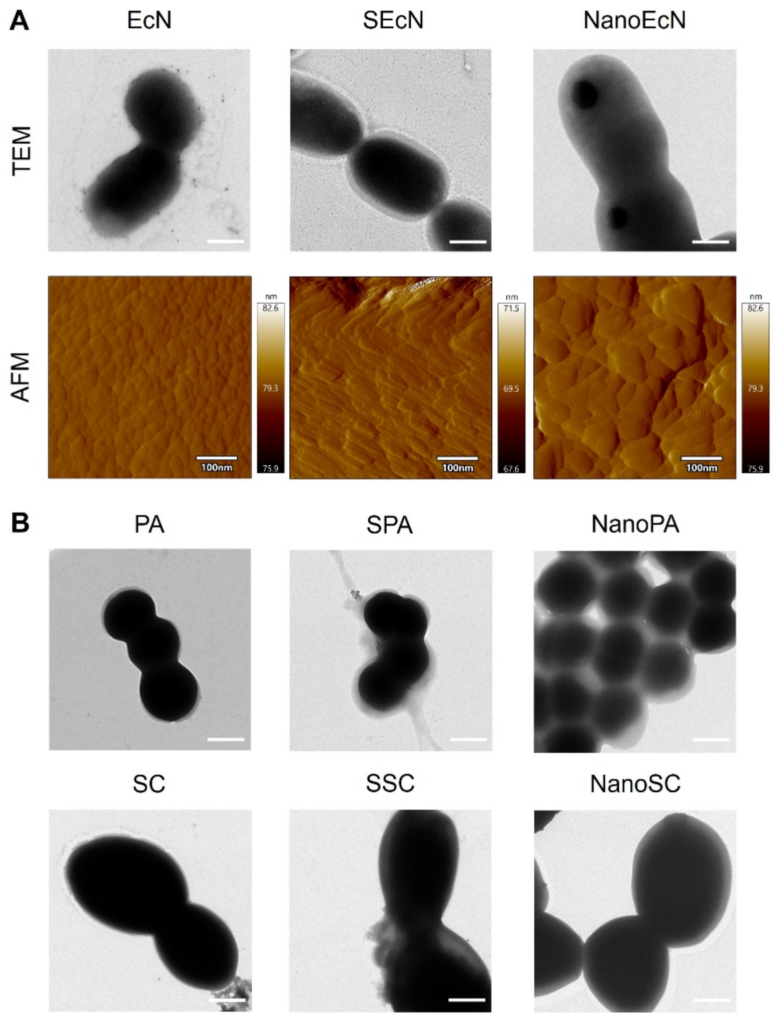

Figure S2 Microscopic images of microbes and their nanoerivatives. (A) TEM and AFM images of EcN and its nanoerivatives. (B) TEM images of PA, SC and nanoerivatives. Scale bar: 500 nm (EcN and PA) or 2 μm (SC).

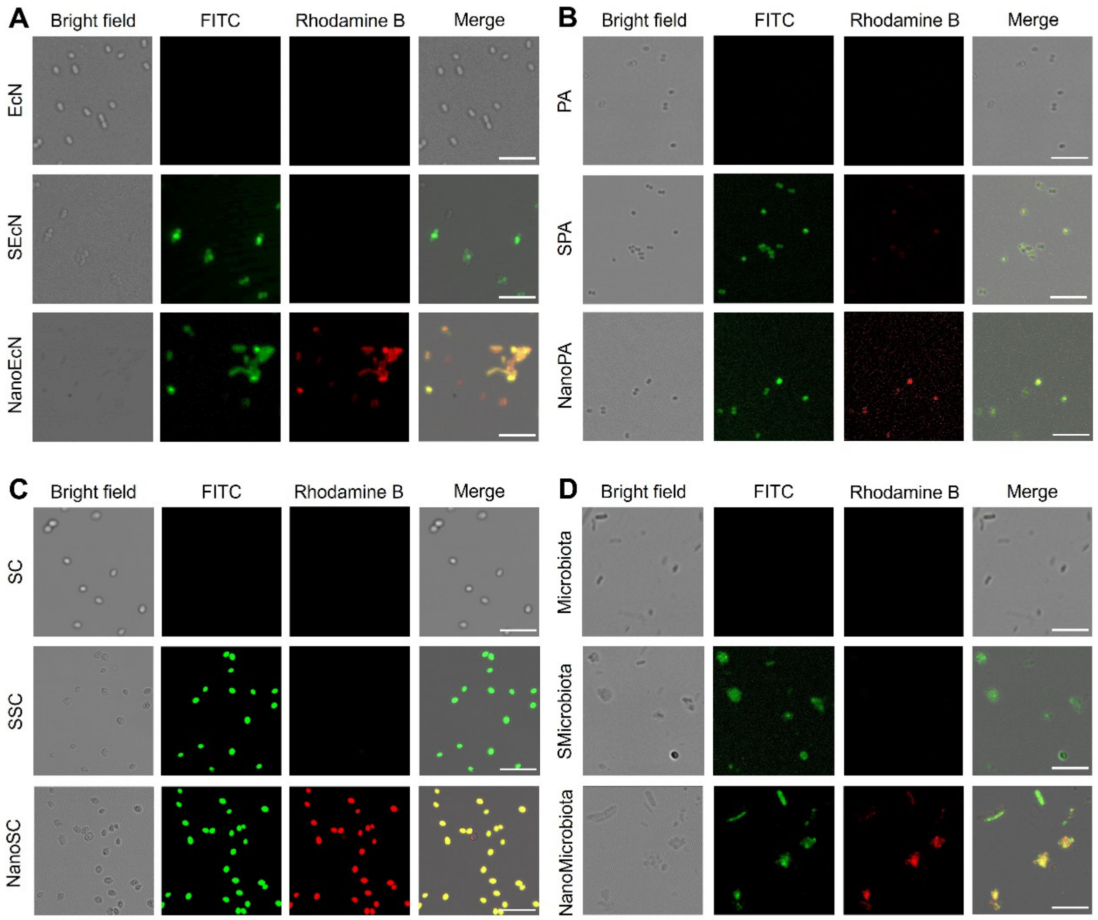

Figure S3 Typical confocal images of microbes and their nanoerivatives. (A) Confocal images of EcN and its nanoerivatives. (B) Confocal images of PA and its nanoerivatives. (C) Confocal images of SC and its nanoerivatives. (D) Confocal images of microbiota and its nanoerivatives. Scale bar: 10 μm or 25 μm (SC).

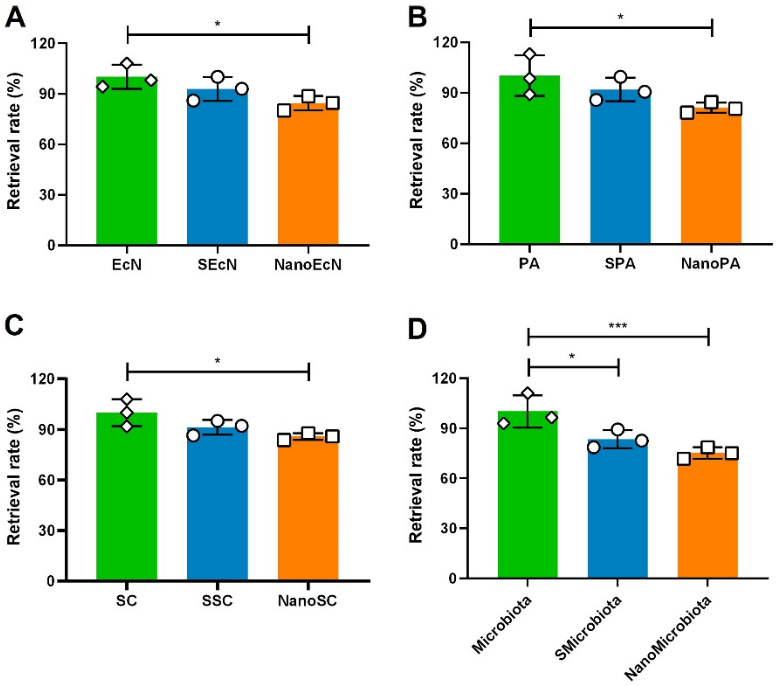

Figure S4 Retrieval rates of microbes during the decorating with silk fibroin and phosphatidylcholine.

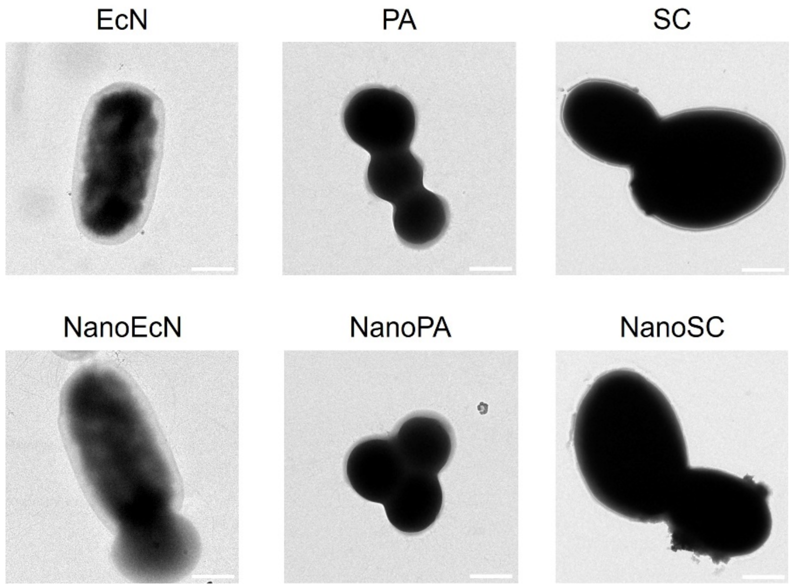

Figure S5 Micromorphological comparison of microbes with their nanoderivatives after 4 h culture at 37 °C and 200 rpm. Scale bar: 500 nm (EcN, NanoEcN, PA and NanoPA) or 2 μm (SC and NanoSC).

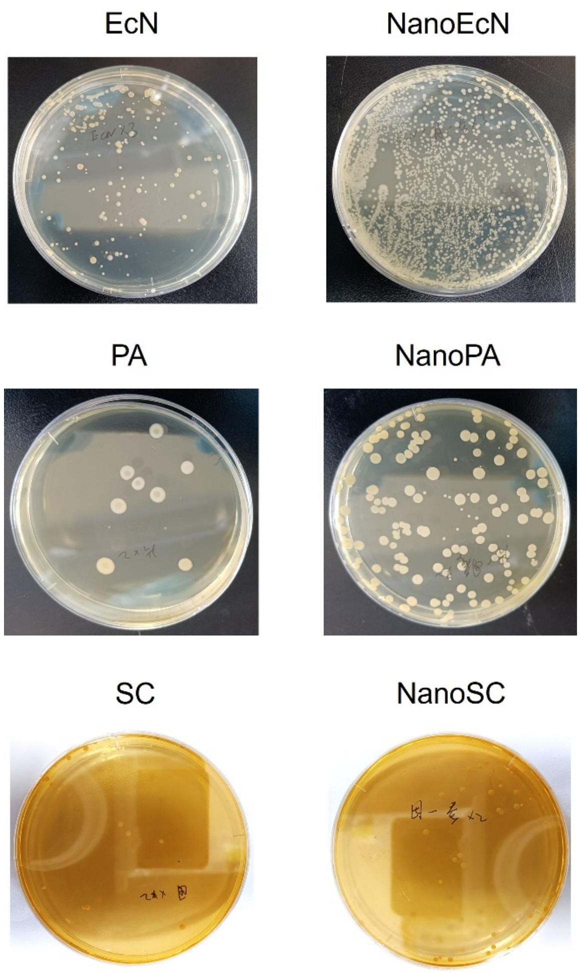

Figure S6 Digital images of colony forming units. EcN, PA, SC, microbiota and their nanoerivatives were exposed to SGF (pH 2.5) supplemented with pepsin for 2 h at 37 ℃ and 200 rpm.

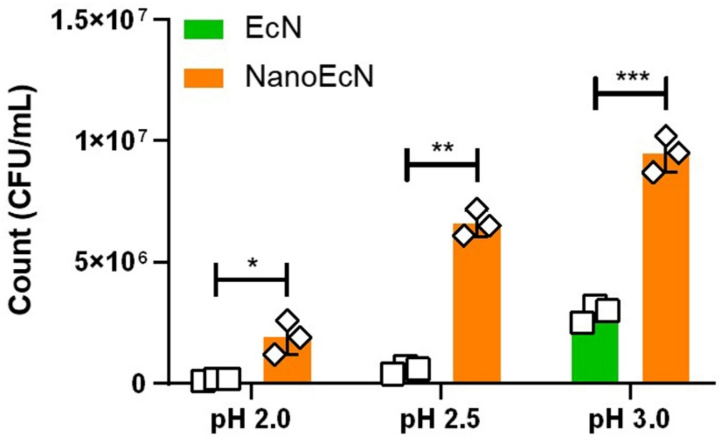

Figure S7 Survival number of nanocapsule-coated EcN after exposure to SGF with different pHs for two hours at 37 °C and 200 rpm.

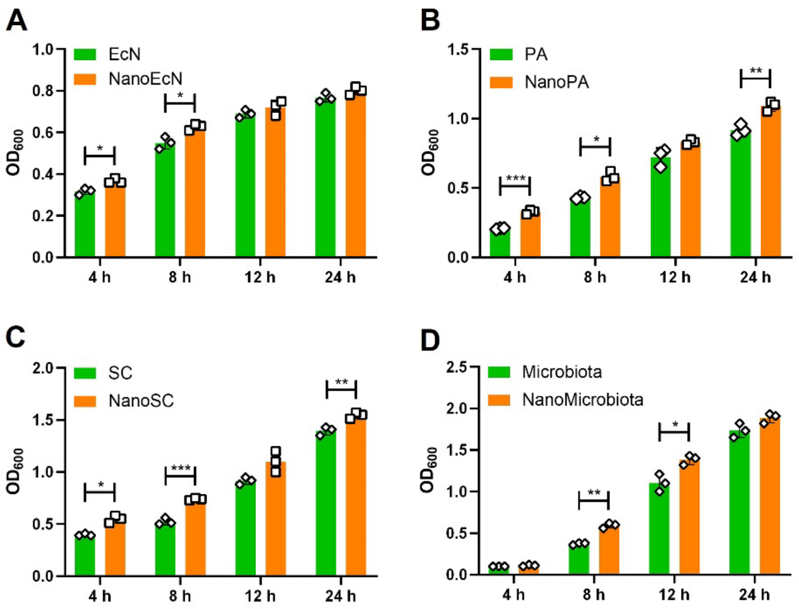

Figure S8 Growth curves of microbes incubated in SIF at 37 °C for 24 h.

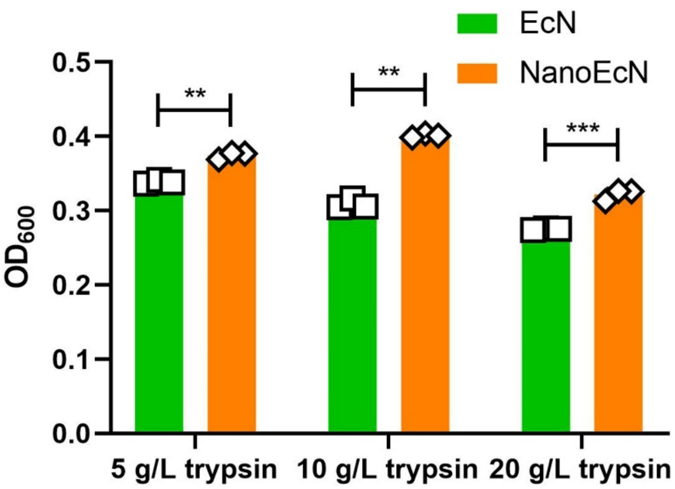

Figure S9 Growth curves of nanocapsule-coated EcN in SIF with different trypsin concentrations for 4 h at 37 ℃.

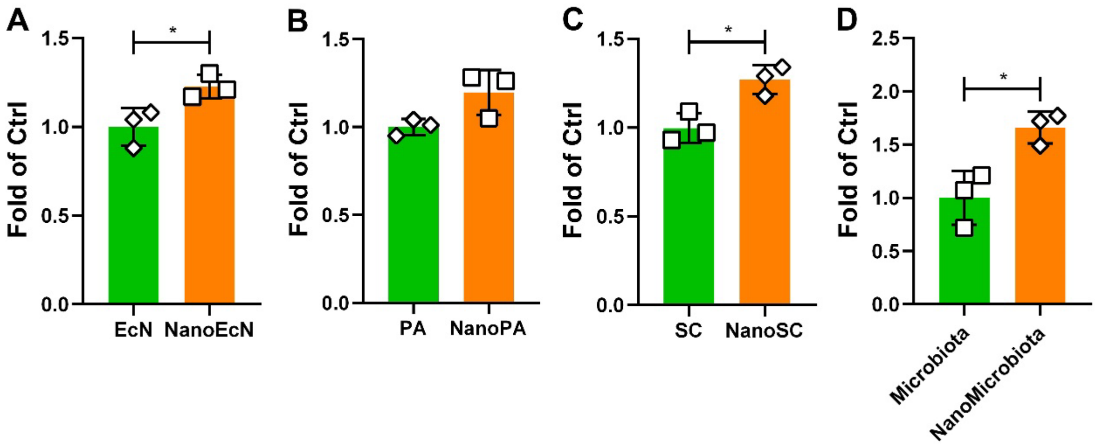

Figure S10 Resistance of microbes after exposure to 0.3 mg/mL CA solution for 4 h at 37 °C and 200 rpm.

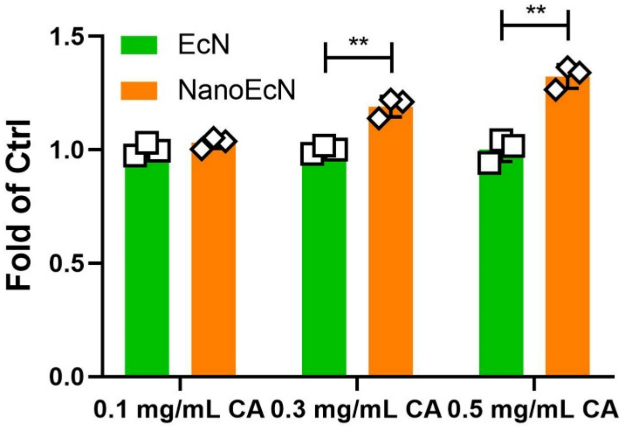

Figure S11 Resistance of nanocapsule-coated EcN after exposure to CA solution for 4 h at 37 °C and 200 rpm.

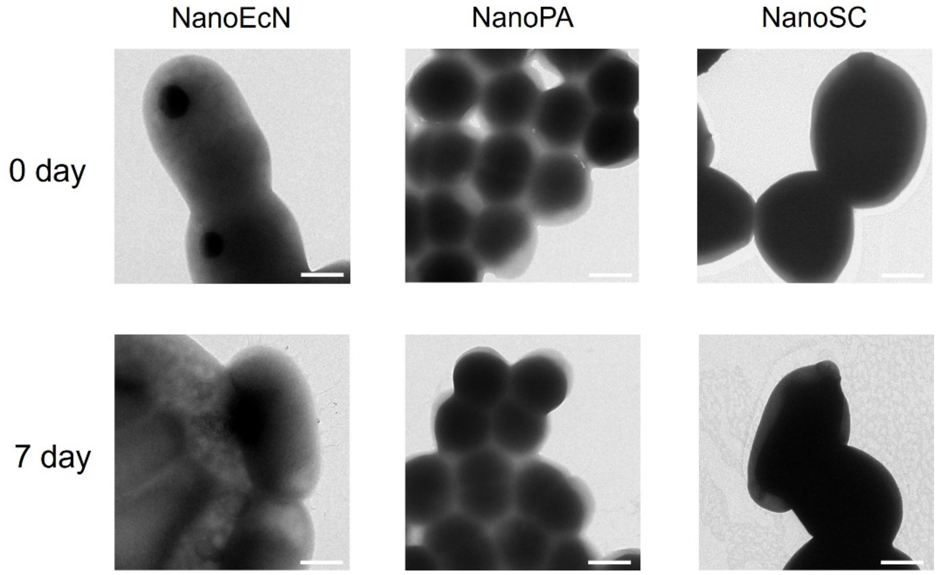

Figure S12 Stability of nanocapsules shown with TEM images after 7 day storage in 4 ℃. Scale bar: 500 nm (EcN, NanoEcN, PA and NanoPA) or 2 μm (SC and NanoSC).

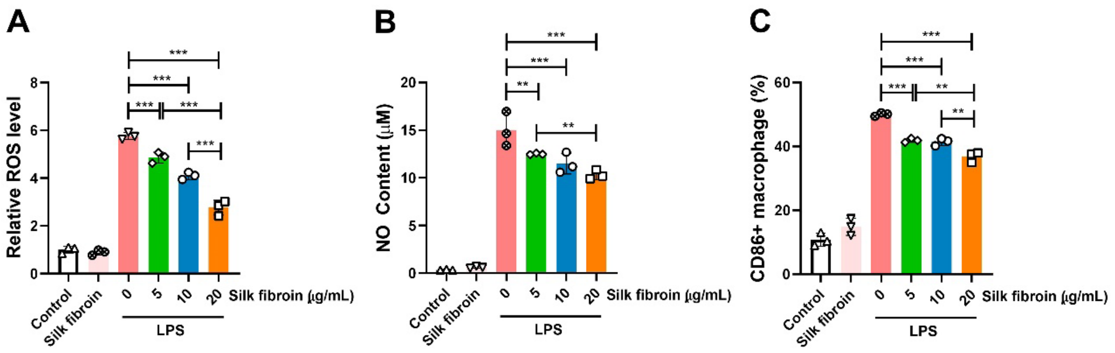

Figure S13 Impact of silk fibroin on ROS level, NO content and percentages of CD86+ macrophages in RAW 264.7 cells.

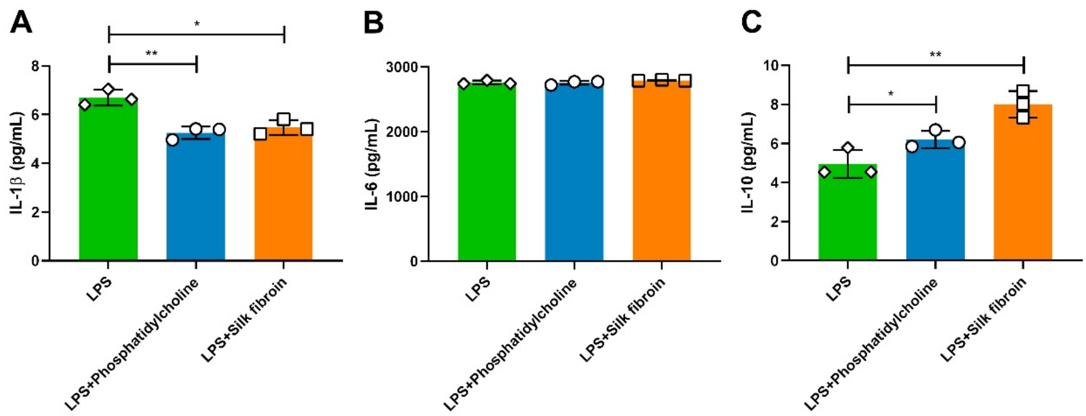

Figure S14 Impact of silk fibroin and phosphatidylcholine on cytokine levels of RAW 264.7 cells after LPS stimulation.

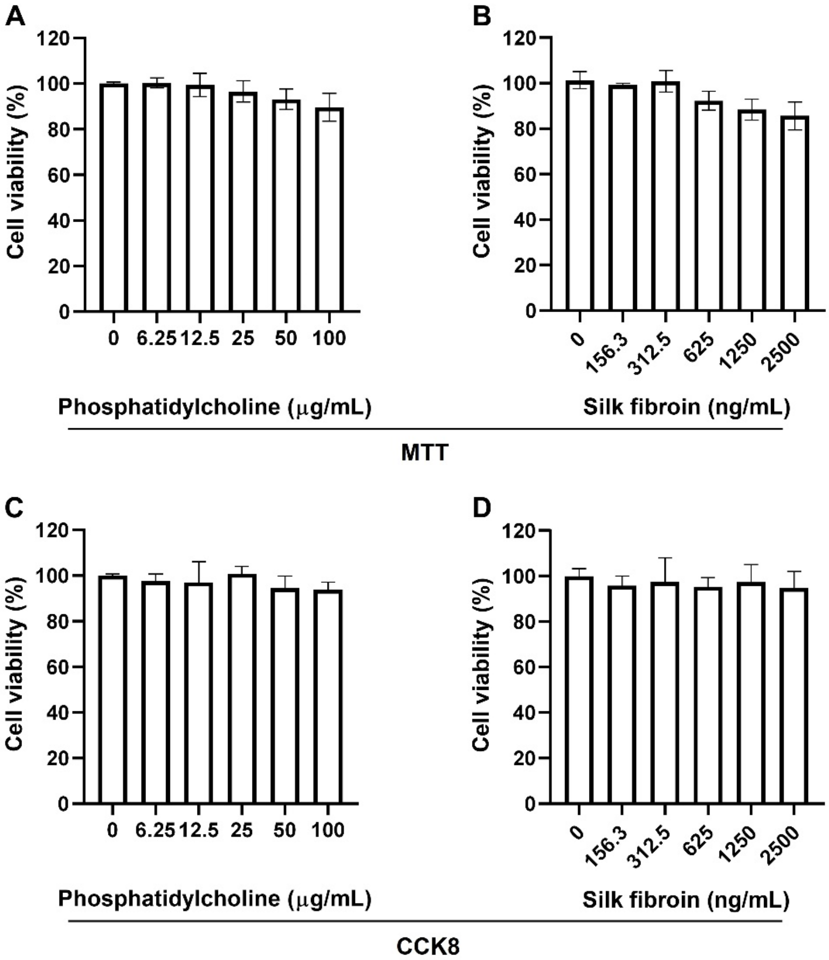

Figure S15 Cell viability of Caco-2 measured with MTT and CCK8 after culture with phosphatidylcholine and silk fibroin for 24 h.

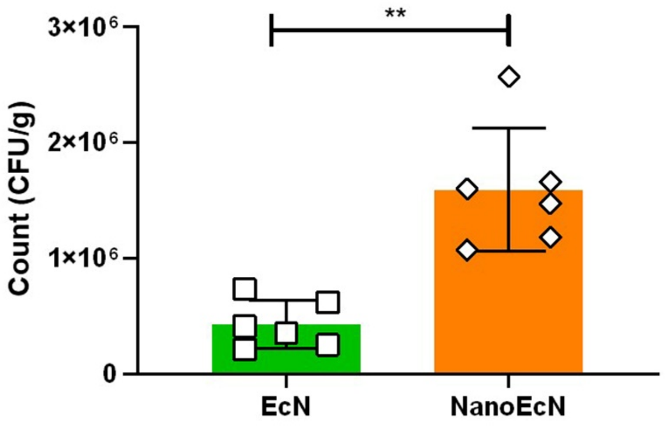

Figure S16 Colonization of EcN in mouse cecum after oral administration using bacterial count of 2×10^7 CFU/mouse on 5 day.

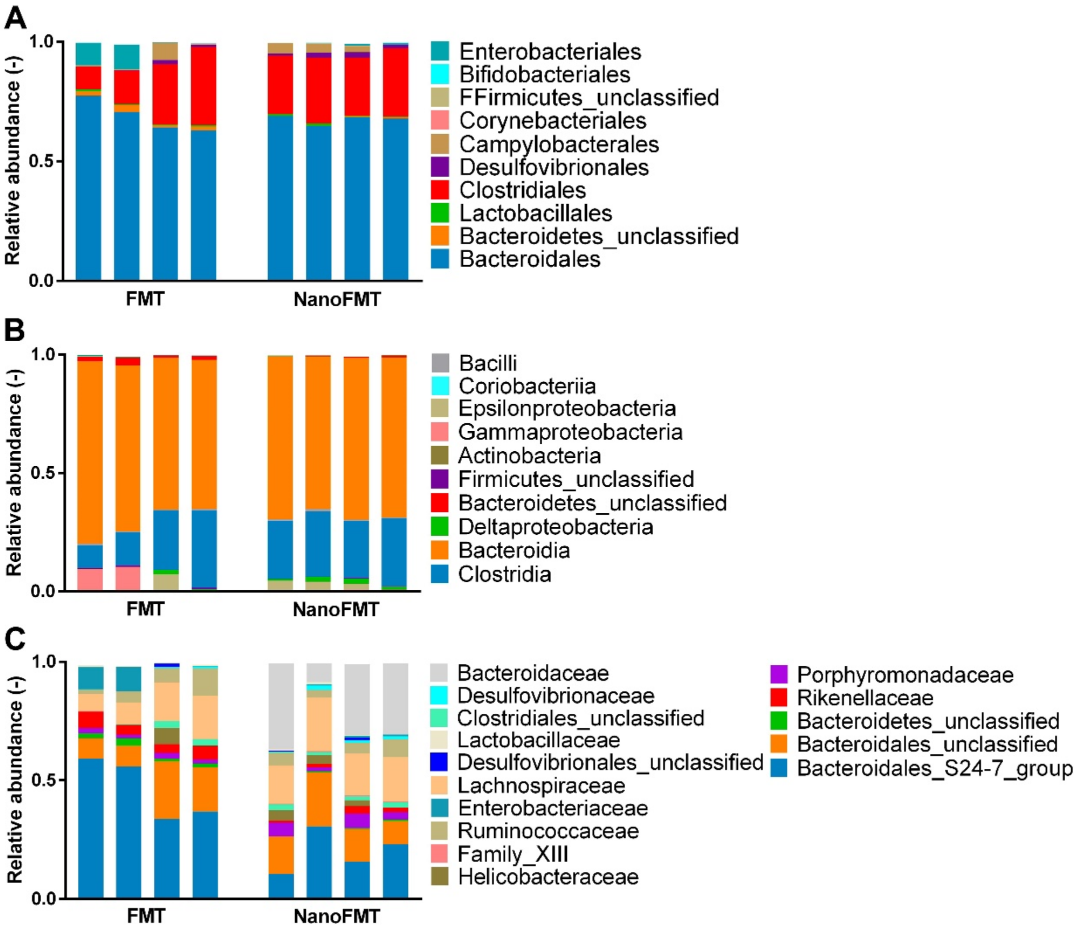

Figure S17 Microbiota abundances of GF mice at order (A), class (B), and family level (C) after NanoFMT.

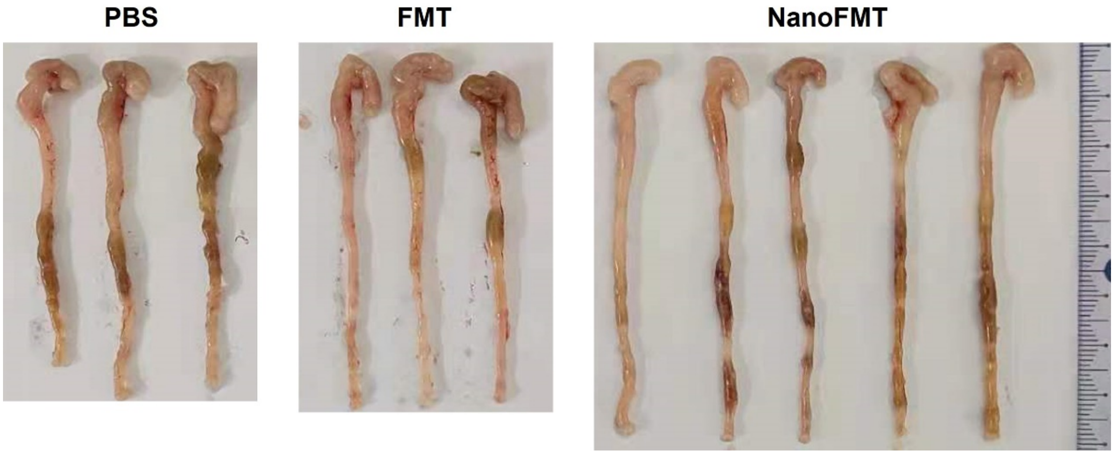

Figure S18 Digital images of colon length in the STm-induced colitis mouse model after treatment.

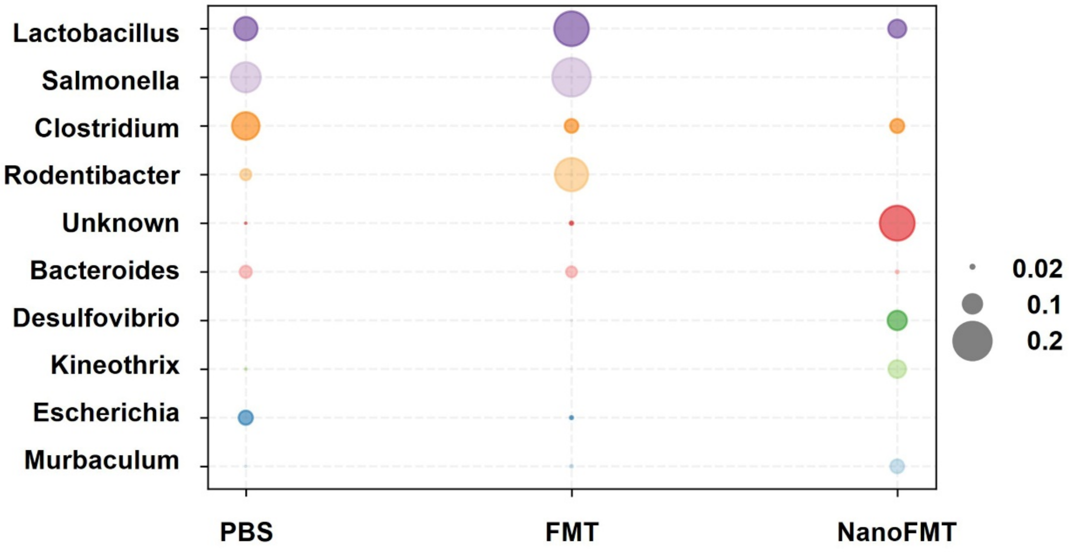

Figure S19 Bubble matrix of gut microbiota at the genus level in the STm-induced colitis mouse model after treatment.

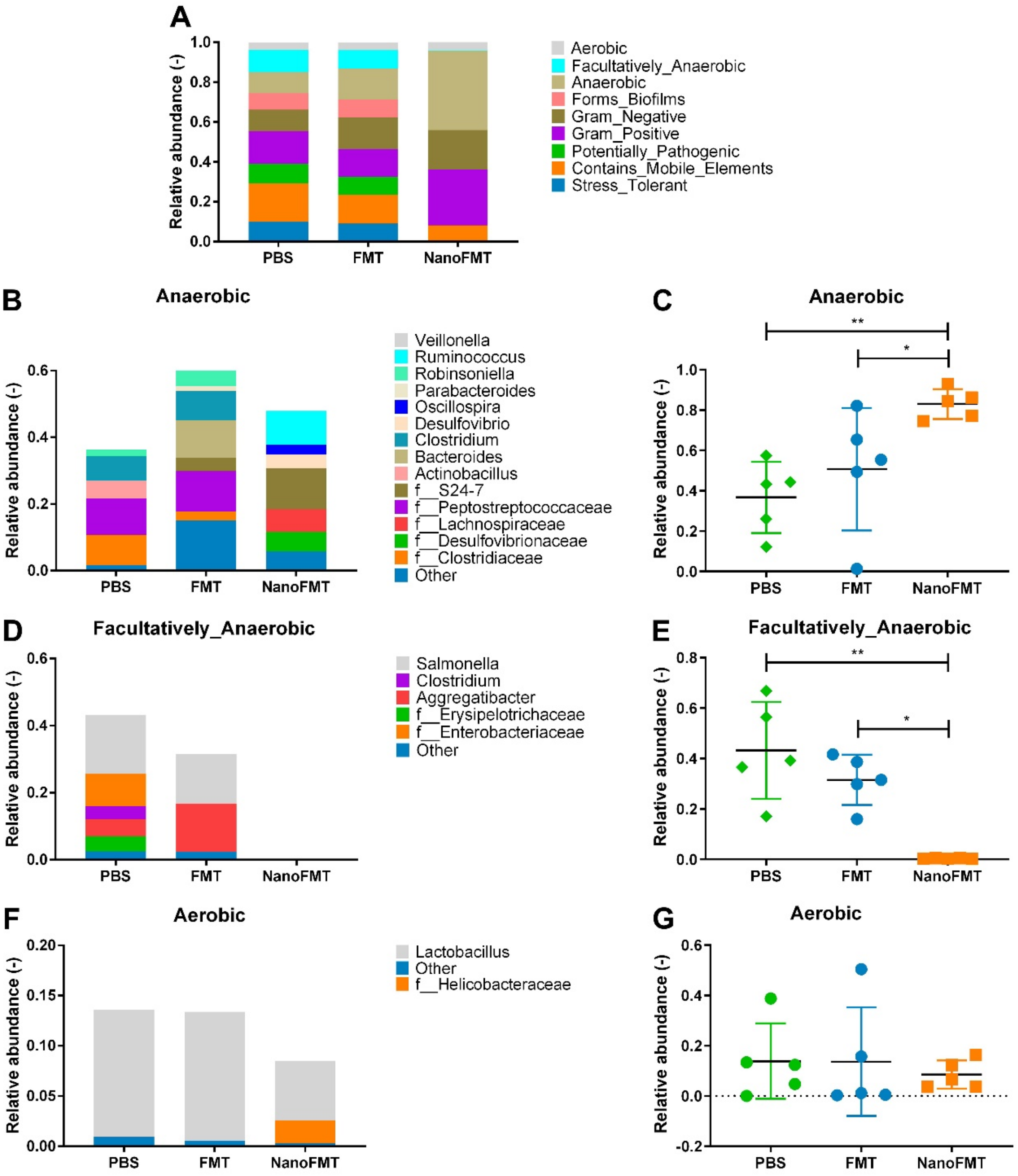

Figure S20 Predicted phenotypes of the gut microbiota in the STm-induced colitis mouse model after treatment. (A) Relative abundance of all phenotypes. (B, C) Relative abundance of anaerobic microbes. (D, E) Relative abundance of facultative anaerobic microbes. (F, G) Relative abundance of aerobic microbes.

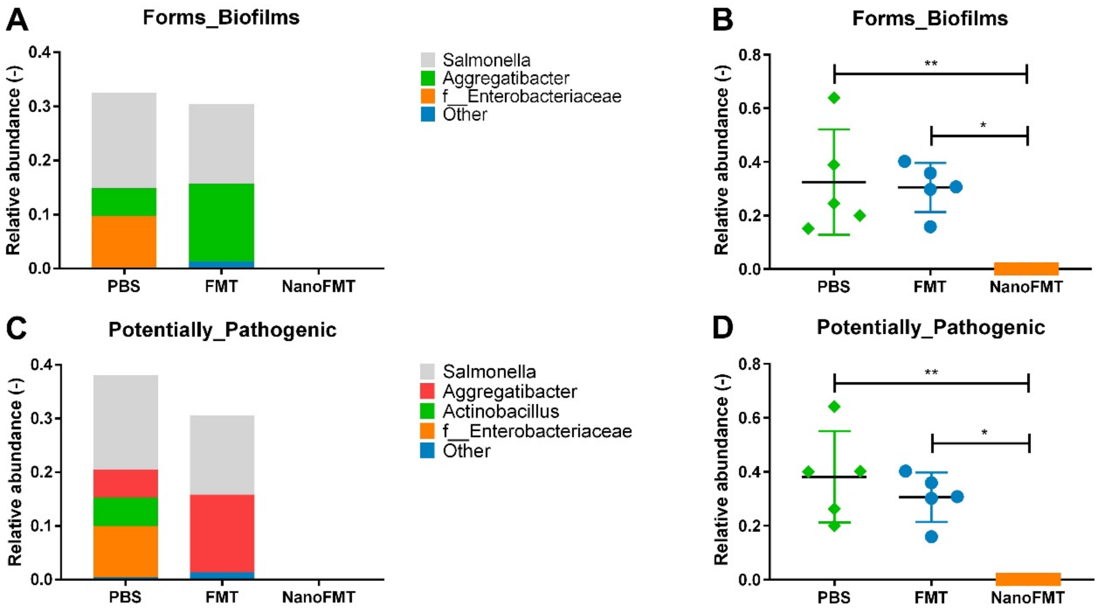

Figure S21 Relative abundance of biofilms formable microbes (A, B) and potentially pathogenic microbes (C, D) in the STm-induced colitis mouse model after treatment.

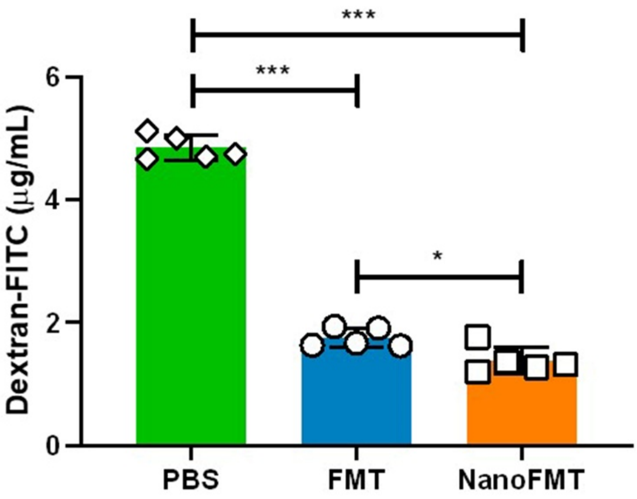

Figure S22 Intestinal permeability of mice after NanoFMT treatment. Fluorescence intensity of serum was measured after gavage 600 mg/kg mouse FITC-dextran.

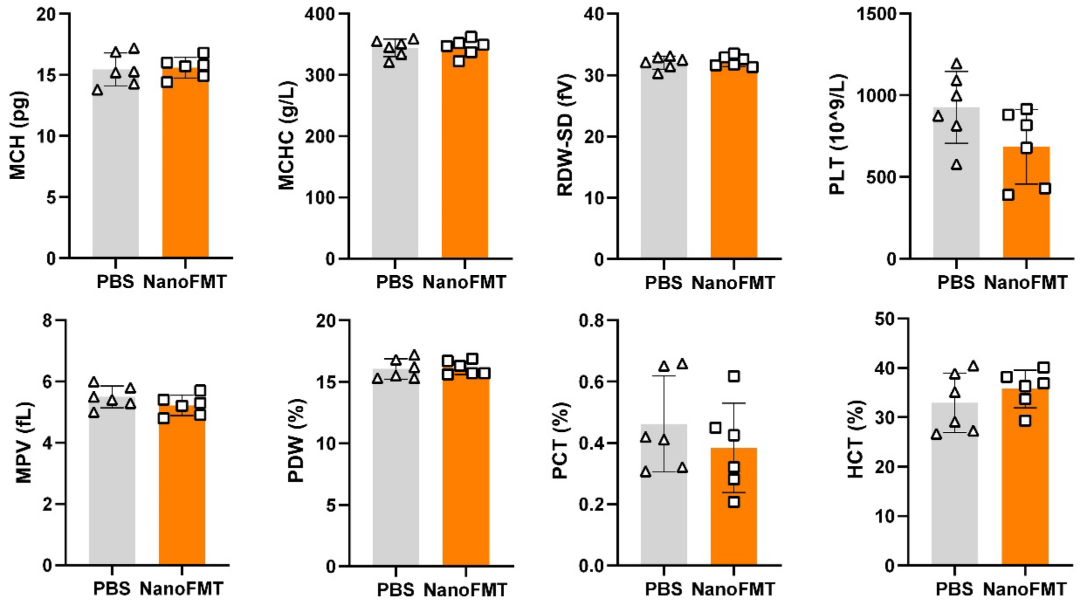

Figure S23 Biosafety evaluation of NanoFMT using hematological analysis.
